# Air Purifiers and Acute Respiratory Infections in Residential Aged Care: A Randomized Clinical Trial

**DOI:** 10.1001/jamanetworkopen.2024.43769

**Published:** 2024-11-11

**Authors:** Bismi Thottiyil Sultanmuhammed Abdul Khadar, Jenny Sim, Vanessa M. McDonald, Julee McDonagh, Matthew Clapham, Brett G. Mitchell

**Affiliations:** 1 School of Nursing and Midwifery, University of Newcastle, Newcastle, New South Wales, Australia; 2 Hunter Medical Research Institute, Newcastle, New South Wales, Australia; 3 School of Nursing, Midwifery and Paramedicine, Australian Catholic University, North Sydney, New South Wales, Australia; 4 School of Nursing, Faculty of Science, Medicine and Health, University of Wollongong, Wollongong, New South Wales, Australia; 5 Centre for Chronic & Complex Care Research, Blacktown Hospital, Western Sydney Local Health District, Blacktown, New South Wales, Australia; 6 School of Nursing, Avondale University, Lake Macquarie, New South Wales, Australia; 7 Nursing and Midwifery, Monash University, Victoria, Australia; 8 Central Coast Local Health District, Gosford, New South Wales, Australia

## Abstract

**Importance:**

The effectiveness of in-room air purification for the reduction of acute respiratory infections (ARIs) in residential aged-care facilities (RACFs) is unknown.

**Objective:**

To investigate the effectiveness of in-room air purifiers with high-efficiency particulate air (HEPA)–14 filters in reducing the incidence of ARIs among residents of RACFs.

**Design, Setting, and Participants:**

This randomized clinical trial used a multicenter, double-blind, 2-period, 2-treatment crossover design for 6 months from April 7 to October 26, 2023, in 3 RACFs with a bed capacity of 50 to 100 in New South Wales, Australia. The purposive sampling approach included permanent residents in private rooms in the enrolled RACFs. Data collection was performed every 2 weeks and required no additional follow-up beyond the final data collection on October 31, 2023.

**Intervention:**

An air purifier containing a HEPA-14 filter was placed in rooms of participants in the intervention group, and an air purifier without a HEPA-14 filter was placed in rooms of the control participants. The groups crossed over after 3 months.

**Main Outcomes and Measures:**

The primary outcome was the incidence of ARIs, assessed with logistic mixed-model regression.

**Results:**

Among 135 participants randomized (70 to the intervention-first group and 65 to the control-first group), 78 (57.8%) were female; mean (SD) age was 85.2 (8.6) years. In the intention-to-treat analysis, the use of air purifiers with HEPA-14 filters did not reduce ARIs compared with the control (OR, 0.57; 95% CI, 0.32-1.04; *P* = .07). Among the 104 participants who completed the entire study, the intervention reduced ARI incidence from 35.6% (37 participants) in the control group to 24.0% (25 participants) in the intervention group (OR, 0.53; 95% CI, 0.28-1.00; *P* = .048).

**Conclusions and Relevance:**

In this clinical trial investigating use of air purifiers with HEPA-14 filters for reducing ARIs, no significant between-group difference was found in the intention-to-treat analysis. However, a significant reduction in ARIs was identified among participants who completed the entire study. These findings may help inform future large-scale studies of respiratory infectious diseases.

**Trial Registration:**

ANZCTR identification: ACTRN12623000347662

## Key Points

**Question:**

In residential aged-care facilities (RACFs), what is the effect of air purification compared with no air purification in the residents’ rooms on the incidence of acute respiratory infections?

**Findings:**

In this randomized clinical trial using a crossover design and including 135 RACF residents, there was no significant difference in the incidence of acute respiratory infections with (25%) vs without (34%) use of air purifiers with high-efficiency particulate air (HEPA)–14 filters.

**Meaning:**

The findings suggest that air purifiers with HEPA-14 filters placed in residents’ rooms do not reduce the incidence of acute respiratory infections among RACF residents.

## Teaser

This crossover randomized clinical trial investigates whether air purifiers with high-efficiency particulate filters reduce the incidence of acute respiratory infections in residential aged-care facilities.

## Introduction

Acute respiratory infections (ARIs), such as influenza virus, SARS-CoV-2, respiratory syncytial virus (RSV), rhinovirus, and adenovirus infections and pneumonia, pose a significant threat to the health of the global community.^1^ The outcomes of ARIs among older adults, particularly those in residential aged-care facilities (RACFs) or long-term care facilities, can be severe.^2^ The COVID-19 pandemic highlighted this and the critical need to better understand effective infection prevention and control strategies.^3,4^

The successful prevention of ARIs requires a thorough understanding of pathogen transmission.^5,6^ Risks of inhalation transmission and subsequent infection are associated with several factors, including pathogen-containing aerosol emission and removal rates, exposure, cumulative dose, and the probability of infection.^5^ One component of a multimodal approach to reducing the risk of ARIs includes clean air, in which air purification may play a role.^6^ Even though there has been a rise in the use of portable air purification devices to prevent ARIs,^7^ the effectiveness of in-room air purification in RACFs for reducing the incidence of ARIs is unknown. We sought to investigate the effectiveness of portable in-room air purifiers with a high-efficiency particulate air (HEPA) filter in reducing the incidence of ARIs among residents of RACFs.

## Methods

### Trial Design

We conducted a multicenter, double-blind, crossover randomized clinical trial (RCT; ACTRN12623000347662) in Australia for 6 months from April 7 to October 26, 2023. A washout period of 1 week occurred between July 14 and 20, 2023. The study phases were selected to align the study period with the historical epidemiologic peak season for influenza in Australia. Participants were followed up every 2 weeks throughout the study phases. Data collection was finalized on October 31, 2023. The 1-week washout period was used between study phases to swap air purifiers between groups and monitor for carryover effects. One week was chosen, as this time frame is consistent with the median incubation period of acute respiratory infections (ARIs), which generally ranges from 1 to 7 days.^8^ Data were not collected during the washout period. Any ARI cases reported during the washout period were not included in the data analysis. We reported findings following the 2010 Consolidated Standards of Reporting Trials (CONSORT) reporting guideline.^9^ A trial protocol has been published elsewhere^10^ and is given in Supplement 1. Ethics approval was granted by the Hunter New England Human Research Ethics Committee, Australia. Written consent was sought from all participants or from the next of kin for participants who were unable to provide informed consent themselves.

### Settings and Participants

The settings were RACFs. Five facilities were initially approached, with the first 3 agreeing to participate as study sites. Sites were eligible if they had over 50 beds, were accredited by the Aged Care Quality and Safety Commission,^11^ and were located in New South Wales (NSW), Australia. Engagement in the study was supported by a senior executive manager in the facilities. The size of these facilities ranged from 50 to 100 beds.

The participants were permanent residents in 1 of the 3 included RACFs in NSW, Australia. A purposive sampling approach based on the researchers’ network was used to invite study sites. All permanent residents in the 3 facilities were invited to participate in the study. Individuals were invited through 1 of 3 methods: email, at the time of provision of written study information, or through discussions with a member of the research team. To be eligible, participants must have been a permanent resident in 1 of the 3 study sites, living in a private room, and not receiving palliative comfort care (trajectory C) at the time of study commencement. No age restrictions were applied. Prior to our study, none of the participating facilities or participants had any mode of existing air purification or filtration system in place. Residents in respite care and those who were under the care of the public guardian were excluded. Potential participants were followed up 3 days after the initial contact to determine whether they wished to consent.

### Intervention

As part of the study, when the participants received the intervention, they had an air purifier containing a HEPA-14 filter (Rediair, GAMA Healthcare) placed in their room. Those receiving the control condition received an identical air purifier that did not contain a HEPA-14 filter. The placement of air purifiers was consistent with the government guideline at the time.^12^ Other zones, including shared or communal areas, were excluded. The size of participants’ rooms was similar between and within facilities (average, 50 m^3^), and all rooms had their own air conditioner. Air purifiers remained at the setting, providing a minimum of 8 air changes per hour. If required, the sleep function could be used at night to reduce lights and noise. This provided a minimum of 3 air changes per hour.^13,14,15^ Supplement 1 provides more information.

### Outcomes

The primary outcome of the study was the incidence of ARIs and was binary: infection or no infection. An ARI was defined as the sudden acute onset of at least 1 of 4 respiratory symptoms, including cough, sore throat, shortness of breath, or coryza, and a clinician’s judgment that the illness was due to an infection.^16,17^ When diagnostic testing was performed and a causative pathogen identified, the primary outcome was further divided into infection with influenza virus (A or B), SARS-CoV-2, rhinovirus, RSV, or adenovirus. Participants did not undergo a diagnostic test as part of the study. Any diagnostic tests undertaken were determined as part of the residents’ routine medical care, facility procedure, or usual practice. Ascertainment bias remained constant throughout the study. The secondary outcomes were time to first infection and numbers of visits to an emergency department, hospital admissions, and medical consultation reviews due to an ARI.

### Sample Size

We assumed the expected proportion of participants with an ARI would be 40%.^18^ With a sample of 94 participants in total, our study would be powered to 80% to identify a 50% reduction in ARI incidence at a 5% significance level. This corresponded to an odds ratio (OR) of 0.38 for air purification (filtration) vs control (no filtration). A 40% attrition rate was estimated, and therefore, a sample of 132 participants was required.

### Randomization, Concealment, and Implementation

Stratified randomization was used. For each site, participants were randomly assigned into 1 of the 2 sequences of intervention and control conditions using a computer-generated randomization process undertaken by a member of the research team (B.G.M.) not involved in the intervention delivery or determination of outcomes. Allocation concealment was ensured, as the researcher responsible for randomization did not disclose the outcome of randomization to any of the research teams and held the allocation in a password-protected database in a file that was not accessible to others. The allocation was disclosed at the point of handing over data to an independent statistician (M.C.) for analysis.

### Blinding

The study was double-blinded. The participants and the researcher (B.T.S.A.K.) collecting data and determining the outcome (whether a participant had an ARI) were unaware for the entirety of the study of whether the participant had an air purifier with a HEPA filter or one without. The research protocol (Supplement 1) provides additional information on sample size calculation, randomization, concealment, implementation, and blinding.

### Data Collection

On-site data collection in participating facilities was carried out every 2 weeks by a member of the research team who was blinded to the intervention allocation (eTable 1 in Supplement 2). Baseline and demographic data for each participant were collected before the commencement of the study. Data on other important variables were also collected, including each resident’s room size; room heating, ventilation, and air conditioning (HVAC) system; and mobility status (eg, whether the participant could mobilize independently; could mobilize with standby assistance, assistance of 1 staff member, or assistance of 2 staff members; or was immobile). A resident’s typical pattern of mobility was also collected by interviewing either the resident or a staff member who was familiar with the resident to document their daily routine. Data on the pattern of mobility were collected before the commencement of phases 1 and 2 of the trial to ensure that changes over time were captured (eTable 2 in Supplement 2). Additional data collection information is provided in eTable 1 in Supplement 2.

### Statistical Analysis

The incidence of ARIs (infection or no infection) was assessed with logistic mixed-model regression. Fixed effects included the intervention, with design-specific variables for each phase and facility. The random effect in the model was the unique participant identification number to account for within-person correlation. Analysis was conducted for all participants, and a subgroup analysis was conducted for participants who completed the study. The secondary outcome of time to first infection was assessed with a Cox proportional hazards regression model with mixed effects (frailty model). Additional information is provided in eTables 12 and 13 in Supplement 2. Statistical analyses were performed using R, version 4.3.2 (R Project for Statistical Computing). Two-sided *P* < .05 was considered significant.

In sensitivity analysis, we sought to understand the impact of missing data. Missing data were treated in 2 ways. For the infection outcome, missing data were treated as missing completely at random by removing the missing data with complete analysis^19^ and as missing at random by imputing those data with multiple imputations. Multiple imputation was conducted with the R package mice^20^ using mixed-effects logistic regression models within chained equations. A total of 100 imputations with 20 iterations were used. The same aforementioned logistic mixed model was fit for each imputation. The 100 estimates were pooled using Rubin rules, and ORs with 95% CIs and *P* values were calculated. Additional sensitivity analysis was undertaken using the same multiple imputation approach without the inclusion of participant demographics (eTables 14 and 15 in Supplement 2).

## Results

### Baseline Characteristics

The study included 135 participants; median age was 86.0 years (range, 59.0 to 103.0 years), and mean (SD) age was 85.2 (8.6) years. The group comprised 78 females (57.8%) and 57 males (42.2%). The baseline characteristics of participants who completed the study were similar to those of the participants who did not complete the study. The baseline data of participants are provided in Table 1.

**Table 1.** Baseline Characteristics of Study Participants

Characteristic	Participants^a^				
	All (N = 135)	Control (n = 65)^b^	Intervention (n = 70)^b^	Completers (n = 104)	Noncompleters (n = 31)
Age, y^c^					
Median (IQR) [range]	86.0 (81.0-91.0) [59.0-103.0]	86.0 (81.0-89.0) [62.0-101.0]	87.0 (80.0-92.0) [59.0-103.0]	85.0 (80.5-91.0) [67.0-103.0]	89.0 (84.0-92.0) [77.0-101.0]
Mean (SD)	85.2 (8.6)	85.0 (8.0)	85.3 (9.3)	84.7 (8.7)	86.9 (8.1)
Sex					
Female	78 (57.8)	41 (52.6)	37 (47.4)	60 (57.7)	18 (58.1)
Male	57 (42.2)	24 (42.1)	33 (57.9)	44 (42.3)	13 (41.9)
MMSE score^d^					
≤24	101 (74.8)	53 (52.5)	48 (47.5)	78 (75.0)	23 (74.2)
≥25	34 (25.2)	12 (35.3)	22 (64.7)	26 (25.0)	8 (25.8)
ACD in place	135 (100)	65 (48.2)	70 (51.9)	104 (100)	31 (100)
History of ARI	13 (9.6)	9 (69.2)	4 (30.8)	10 (9.6)	3 (9.7)
Specific medications^e^	13 (9.6)	7 (53.9)	6 (46.2)	12 (11.5)	1 (3.2)
Vaccination status					
Influenza	127 (94.1)	63 (49.6)	64 (50.4)	98 (94.2)	29 (93.6)
COVID-19	127 (94.1)	61 (48.0)	66 (52.0)	101 (97.1)	26 (83.9)
Pneumococcal pneumonia	31 (23.0)	14 (45.2)	17 (54.9)	28 (26.9)	3 (9.7)
Mobility status					
Immobile^f^	32 (23.7)	13 (40.6)	19 (59.4)	23 (22.1)	9 (29.0)
Assistance by 2 persons	21 (15.6)	9 (42.9)	12 (57.1)	18 (17.3)	3 (9.7)
Assistance by 1 person	23 (17.0)	12 (52.2)	11 (47.8)	19 (18.3)	4 (12.9)
Standby assistance^g^	9 (6.7)	6 (66.7)	3 (33.3)	6 (5.8)	3 (9.7)
Independent^h^	50 (37.0)	25 (50.0)	25 (50.0)	38 (36.5)	12 (38.7)
In-room ventilation					
Can open window	49 (36.3)	26 (53.1)	23 (46.9)	39 (37.5)	10 (32.3)
Own AC unit in room	135 (100)	65 (48.2)	70 (51.9)	104 (100)	31 (100)
In-room HVAC connected to other rooms	0	0	0	0	0

Abbreviations: AC, air conditioning; ACD, advance care directive; ARI, acute respiratory infection; HVAC, heating, ventilation, and air conditioning; MMSE, Mini-Mental State Examination; NA, not applicable.

^a^ Data are presented as number (percentage) of participants unless otherwise indicated. Percentages in the control and intervention columns were calculated with the row totals as denominators, and percentages in the all, completer, and noncompleter columns were calculated with the column totals as denominators.

^b^ Phase 1 characteristic (ie, treatment first or control first).

^c^ At baseline.

^d^ Score range, 0 to 30, with a score of 23 or lower indicative of dementia.

^e^ Antibiotic, antiviral, cytotoxic, or other systemic medications.

^f^ Device and staff aided moving.

^g^ Required close supervision and standby assistance.

^h^ Could move freely unaided.

Between February 4 and March 6, 2023, a total of 223 residents from the enrolled RACFs were screened for eligibility per the research protocol (Figure and Supplement 1). Overall, 148 residents initially consented to be part of the study. Prior to study commencement, 13 participants dropped out (9 withdrew consent, and 4 commenced a palliative care pathway). At study commencement, 135 participants were randomly allocated (70 to the intervention group and 65 to the control group). The allocation of participants to each group within each facility is presented in eTable 3 in Supplement 2.

**Figure.**
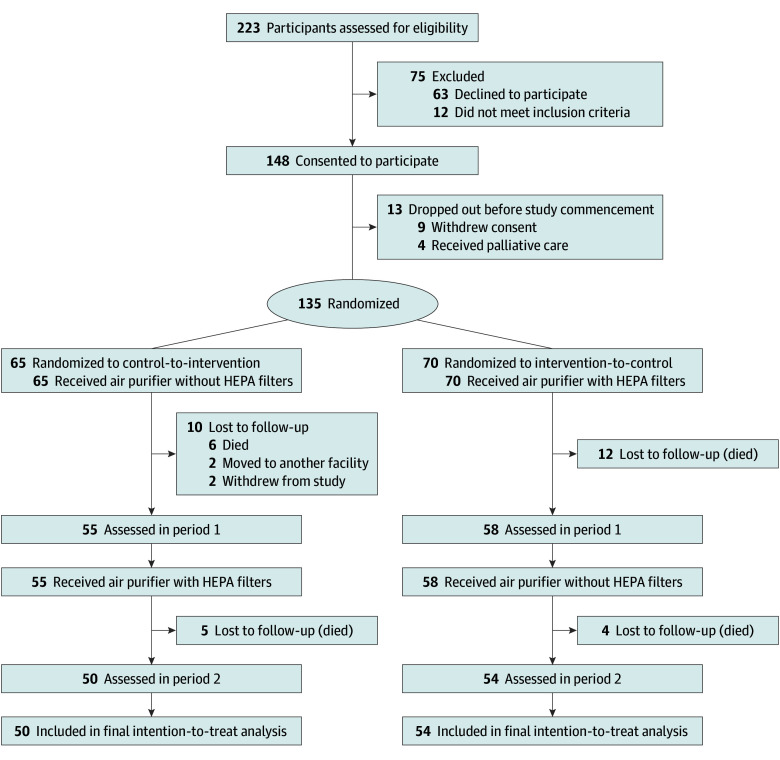
CONSORT Diagram of Study Participants HEPA indicates high-efficiency particulate air.

### Primary Outcome

Of the 135 participants who commenced phase 1, 113 (83.7%) remained in the study at the beginning of phase 2. The proportion of participants with an ARI in each phase of the study, stratified by control and intervention arms, is presented in Table 2. In the first phase of the study, it was identified that 5 participants (3.7%) had multiple ARIs; this increased to 9 (6.7%) in the second phase. Most ARIs (37 of 73 [50.7%]) did not have a causative pathogen identified. Among the 36 ARIs with an identified pathogen, the causative pathogens were SARS-CoV-2 (19 [52.8%]), RSV (16 [44.4%]), and rhinovirus (1 [2.8%]) (eTable 4 in Supplement 2).

**Table 2.** Number of ARIs in the Entire Participant Group and the Study Completers Throughout the Study Period

Study phase	Participants, No./total No. (%) [95% CI]		
	Total	Control	Intervention
**All participants** ^a^			
1	50/135 (37.0) [29.2-45.4]	29/65 (44.6) [32.9-56.8]	21/70 (30.0) [20.2-41.5]
2	23/113 (20.4) [13.7-28.5]	13/58 (22.4) [13.1-34.5]	10/55 (18.2) [9.6-30.0]
Overall	NA	42/123 (34.2) [26.2-42.9]	31/125 (24.8) [17.8-32.9]
**Participants who completed the study**			
1	40/104 (38.5) [29.5-48.1]	24/50 (48.0) [34.5-61.8]	16/54 (29.6) [18.6-42.8]
2	22/104 (21.2) [14.1-29.8]	13/54 (24.1) [14.1-36.8]	9/50 (18.0) [9.2-30.5]
Overall	NA	37/104 (35.6) [26.8-45.1]	25/104 (24.0) [16.7-32.9]

Abbreviations: ARI, acute respiratory infection; NA, not applicable.

^a^ Includes participants lost to follow-up.

#### All Participants

There were 42 ARIs among the 123 participants in the total control group (34.2%; 95% CI, 26.2%-42.9%) compared with 31 ARIs among 125 participants in the total intervention group (24.8%; 95% CI, 17.8%-32.9%) (Table 2). There was no significant difference in incidence of ARIs between the groups (OR, 0.57; 95% CI, 0.32-1.04; *P* = .07) (Table 3).

**Table 3.** Logistic Regression Models for Risk of Acute Respiratory Tract Infection

Characteristic	All participants (n = 135)^a^		Study completers (n = 104)^b^	
	OR (95% CI)	*P* value	OR (95% CI)	*P* value
Treatment				
Control	1 [Reference]	NA	1 [Reference]	NA
Intervention	0.57 (0.32-1.04)	.07	0.53 (0.28-1.00)	.048
Phase				
1	1 [Reference]	NA	1 [Reference]	NA
2	0.37 (0.20-0.69)	.002	0.40 (0.21-0.76)	.006
Site				
A	1 [Reference]	NA	1 [Reference]	NA
B	1.24 (0.54-2.82)	.61	1.29 (0.55-3.05)	.56
C	0.84 (0.43-1.65)	.61	0.70 (0.34-1.42)	.32

Abbreviations: NA, not applicable; OR, odds ratio.

^a^ Phase 1 had 121 participants and phase 2, 105 participants.

^b^ Phases 1 and 2 had 104 participants.

In phase 1, there were significantly more ARIs in the control group (29 among 65 participants [44.6%; 95% CI, 32.9%-56.8%]) compared with the intervention group (21 among 70 participants [30.0%; 95% CI, 20.2%-41.5%]) (Table 2). In phase 2, there was no significant difference in ARIs between the control and intervention groups (Table 2).

#### Subgroup Analysis of Participants Who Completed the Study

A total of 104 participants (77.0%) completed the entire study and were exposed to both the control and the intervention. Thirty-one participants (23.0%) did not complete the entire study (eTable 5 in Supplement 2). The details of outcomes for participants with or without an ARI who dropped out of the study are shown in eTable 6 in Supplement 2. Of those who did not complete the study, 27 (87.1%) died, 2 (6.5%) were transferred to another facility, and 2 (6.5%) withdrew from the study. The study locations and outcomes for completers of the study are presented in eTable 7 in Supplement 2.

Sixty-two ARIs were reported among the 104 participants who completed the study (29.8%). There were 37 ARIs among these 104 participants when exposed to the control (35.6%; 95% CI, 26.8%-45.1%), compared with 25 among the 104 participants when exposed to the intervention (24.0%; 95% CI, 16.7%-32.9%) (Table 2). There were significantly fewer ARIs among participants when exposed to the intervention (OR, 0.53; 95% CI, 0.28-1.00; *P* = .048) (Table 3). The proportions of infections among study completers by phase, intervention, and site are provided in eTable 16 in Supplement 2 and are presented visually in eFigure 1 in Supplement 2.

### Secondary Outcomes

There was no reduction in time to first ARI among participants receiving the intervention vs the control (hazard ratio [HR], 0.67; 95% CI, 0.42-1.07; *P* = .09) (eTable 8 in Supplement 2). We used a novel approach, restricted mean time, in our analysis, which is different from the mean time to infection and allowed an accurate measurement of the survival time of all participants, including those who dropped out.^21^ The restricted mean (SE) time to infection within 90 days was 74.2 (2.3) days in the control period and 78.1 (2.1) days in the intervention period (eTable 9 in Supplement 2). In participants who completed the entire study, the time to first ARI was not reduced among participants receiving the intervention vs the control (HR, 0.62; 95% CI, 0.37-1.02; *P* = .06) (eTable 8 in Supplement 2). Full models of binary outcome variables (infection or no infection) are presented in eTable 8 in Supplement 2. The survival curves are presented in eFigure 2 in Supplement 2.

Among the 73 participants with an ARI, 55 (75.3%) required a medical consultation for the management of their ARI and 10 (13.7%) required an emergency department or nurse practitioner presentation. A total of 10 of these participants (13.7%) were admitted to the hospital for treatment of an ARI (eTable 10 in Supplement 2).

### Sensitivity Analysis

After accounting for missing data using multiple imputations, we found no reduction in the odds of an ARI in the intervention group (OR, 0.56; 95% CI, 0.30-1.03; *P* = .06) (eTable 15 in Supplement 2). Additional data related to sensitivity analysis are given in eTable 11 in Supplement 2. Data from the multiple imputation model, which excluded participant demographics, are presented in eTables 14 and 15 in Supplement 2.

## Discussion

We sought to examine the effectiveness of in-room (portable) air purification for reducing the incidence of ARI for residents in RACFs. We found no reduction in ARIs among participants exposed to air purification using a HEPA filter for the entire population group studied, noting that uncertainty remains given the variation in the incidence of ARIs between phases. More ARIs were identified in phase 1 compared with phase 2, and in phase 2, no differences were identified. We planned for the washout period of our study to coincide with the usual, historical epidemiologic peak of influenza season.^22^ During the course of this study, the number of influenza-like illness notifications peaked earlier than predicted in June 2023,^22^ corresponding to the midpoint of phase 1. In a subanalysis in which data from only the participants who completed the entire study were analyzed, we identified a significant reduction in ARIs among participants exposed to the intervention. Our secondary analyses also identified some important findings—in particular, the proportion of participants requiring a medical or nurse practitioner consultation (75.3%) and the number of residents required to transfer to an emergency department. Hence, it could be argued there was a clinically important reduction in ARIs as an effect of the intervention.

We also considered several other elements that could have potentially influenced our study. The data on the time that participants spent inside their rooms were collected before the commencement of each phase of the trial and did not vary significantly across the periods. Vaccination status and compliance with face masks were not measured as part of the study data. However, during the entire study, all visitors and staff at the participating facilities, including the research team, were required to wear a surgical mask (as a minimum) at all times.^23^ A rapid antigen test negative for SARS-CoV-2 and up-to-date vaccination against SARS-CoV-2 and influenza were verified before every entry to the facility.^23^ All of these measures were mandated and enforced.

The enrolled facilities had no preexisting HVAC system available in resident rooms. Natural ventilation by opening a window to the outside was available in all rooms.

### Strengths and Limitations

Our study has several strengths. Our study provides a rationale for a large, multicenter cluster RCT that incorporates air purification at a cluster level to assess the health benefits at the organizational level. We chose in-room air purification due to the proliferation of air purifiers available for purchase and the difficulty of retrofitting existing RACFs with air purification. Our study was conducted in a clinical scenario; thus, our results are reflective of the complexities of human behavior and the potential effect this could have on outcomes. We chose a crossover design to enable participants to act as their own control, thus accounting for important confounders such as, but not limited to, vaccination, exposure to others, and activities of daily living. During the course of the study, we monitored for changes in these variables and found no noticeable differences (Table 1 and eTables 1 and 2 in Supplement 2). We conducted the study at a time when daily rapid antigen testing (with a negative result) and assessment of fever were requirements for entry to the facility, in addition to the mandatory use of face masks. Double-blinding and our concealment processes were aimed at reducing bias associated with observational studies.

We also recognize study limitations. Given the target population, a dropout rate was not unexpected, but it did have an important implication. We transparently reported all available results to explore differences. Our study was limited to in-room air purification, and the results should not be interpreted beyond this. In addition, the selection of study commencement in April so that phase 1 ended at the historical epidemiologic peak of ARIs in the Australian winter may have had a confounder effect on the study outcome.

## Conclusions

In this crossover RCT, the use of in-room air purifiers with HEPA-14 filters did not result in a reduction in ARIs among RACF residents using an intention-to-treat analysis. The data analysis was challenged with missing data due to participant dropouts, which were expected in this population. The study was designed as a pragmatic trial focused on the effect of the intervention in a residential aged care setting. Although the study results were not statistically significant, they may be clinically important. A reduction in the absolute number of ARIs among participants using the intervention who completed the study was identified. The findings from this study may help inform future large-scale RCTs in respiratory infectious diseases by contributing to a quality health care system framework.

## Notes

Supplement 1.
Trial Protocol

Supplement 2. **eTable 1.** Fortnightly Routine Data Collection Related to an ARI **eAppendix 1.** Additional Trial Information **eTable 2.** Data Related to the Mobility Pattern of Study Participants Over the 2 Phases of the Trial **eTable 3.** Allocation of Participants by Study Sites **eTable 4.** Types of Acute Respiratory Tract Infection by Study Period and Intervention Allocation **eTable 5.** Completion Status by Intervention Allocation **eTable 6.** Outcomes for Participants Who Dropped Out of the Study **eTable 7.** Study Location and Outcomes From Participants Who Completed the Study **eAppendix 2.** Secondary Outcomes **eTable 8.** Risk of Infections—Cox Proportional Hazards Regressions for Complete and all Available Cases of Infections **eTable 9.** Mean Days to First Infection **eTable 10.** Other Outcomes in Participants Who Experienced an ARI **eAppendix 3.** Sensitivity Analysis **eTable 11.** Risk of Infections, Phase 1 and 2 Separately **eTable 12.** Risk of Infections—Cox Proportional Hazard Regressions for Complete and all Available Cases of Infections **eTable 13.** Risk of Infection, Phase 1—Cox Proportional Hazard Regressions for Complete and All Available Cases of Infections **eAppendix 4.** Multiple Imputation Model **eTable 14.** Risk of Acute Respiratory Tract Infection—Logistic Regression Models Without Multivariate Adjustments for the Multiple Imputation Model **eTable 15.** Risk of Acute Respiratory Tract Infection—Logistic Regression Models **eTable 16.** Frequency and Percentage of Acute Respiratory Tract Infection by Phase, Intervention, and Site Among Study Completers **eFigure 1.** Proportion of Infections by Phase, Intervention, and Site for Completers **eFigure 2.** Survival Curves for Infections, Stratified by Treatment and Phase **eAppendix 5.** Considerations for Future Studies
eReferences

Supplement 3.
Data Sharing Statement
