# Error in Funding/Support

**DOI:** 10.1001/jamanetworkopen.2024.52013

**Published:** 2024-12-09

In the Original Investigation titled “Air Purifiers and Acute Respiratory Infections in Residential Aged Care: A Randomized Clinical Trial,”^1^ published November 11, 2024, RTP funding provided by grant GNT 2008392 from the University of Newcastle, Australia, was erroneously included in the Funding/Support and has been removed.
