# Supplementary material for: Air Purifiers and Acute Respiratory Infections in Residential Aged Care: A Randomized Clinical Trial
Source: JAMA Netw Open. 2024 Nov 11;7(11):e2443769. doi: 10.1001/jamanetworkopen.2024.43769 (PMC11555545; doi:10.1001/jamanetworkopen.2024.43769)
Supplement: Supplement 1. — Trial Protocol [file jamanetwopen-e2443769-s001.pdf]

Research Protocol

Identifying the effect of air purifiers in reducing the incidence of Acute Respiratory Infections (ARIs) among residents in Australian Residential Aged Care Facilities (RACFs): A Randomised Control Trial

Version 2.0

Ph.D. Candidate
Bismi Thottiyil Sultanmuhammed Abdul Khadar
Student Number: c3399950
Email: Bismi.ThottiyilSultanmuhammedAbdul@uon.edu.au

Supervisors
Associate Professor Jenny Sim (email: jenny.sim@newcastle.edu.au)
Professor Brett Mitchell (email: brett.mitchell@avondale.edu.au)
Dr Julee McDonagh (email: julee.mcdonagh@newcastle.edu.au)

DATE: 24/01/2023

This document contains confidential information and may be subject to confidentiality obligations imposed by legislation or subject to intellectual property protection or copyright. Except as otherwise agreed to in writing, any use, disclosure, copying, or distribution of this document is prohibited.

Contents

Acronyms and Abbreviations 4
1. Version control 5
2. Administrative information 6
2.1 Title 6
2.2 Trial registration 6
2.3 Funding, sponsors, and partners 6
3. Study team and governance 7
Study Team 7
Governance 7
4. Background 8
5. Methods 9
5.1 Aim 9
5.2 Design 9
5.3 Population and recruitment 10
Study setting 10
Facility enrolment 10
Participant recruitment 10
Participants' eligibility Criteria 11
Inclusion criteria 11
Exclusion criteria 12
5.4 Enrolment 12
Individual participants 12
Delays 12
Discontinuation of study or study site 13
5.5 Intervention 13
Fidelity 14
5.6 Outcomes 15
Primary outcome 15
Secondary outcomes 16
5.7 Sample size 16
5.8 Randomisation and concealment 18
5.9 Blinding 18
5.10 Data collection 19
5.11 Data analysis 24
Primary outcome 24

Secondary outcome....25
5.12 Confounders....25
6. Monitoring....25
6.1 Safety evaluations....26
6.2 Adverse event reporting....26
6.3 Incident monitoring and reporting....27
7. Consent....27
7.1. Overview....27
8. Data Management....28
8.1 Privacy and data management....28
8.2 Confidentiality and data management....29
8.3 A plan for making information arising from the research available....30
8.4 Possibility of commercial exploitation....30
9. Human Research Ethics....30
9.1 Overview....30
9.2 Confidentiality....31
10. Dissemination....31
11. Communication of findings to participants....31
12. References....33

Acronyms and Abbreviations

Abbreviation	Meaning
ACQSC	Aged Care Quality and Safety Commission
AHPRA	Australian Health Practitioner Regulation Authority
ANZCTR	Australia New Zealand Clinical Trial Registry
ARIs	Acute Respiratory Infections
CReDITSS	Clinical Research Design & Statistics
ECDC	European Centre for Disease Prevention and Control
HEPA	High-Efficiency Particulate Absorbing
HNE	Hunter New England
HREC	Human Research Ethics Committee
IPC	Information and Privacy Commission
MMSE	Mini-Mental State Examination
NSAF	National Screening and Assessment Form
NHMRC	National Health and Medical Research Council
RACFs	Residential Aged Care Facilities
RTP	Research Training Program
RCT	Randomised Control Trial
REGIS	Research Ethics and Governance Information System
RSV	Respiratory Syncytial Virus
RT-PCR	Reverse Transcription-(qualitative) polymerase chain reaction
SARI	Severe Acute Respiratory Infections
TGA	Therapeutic Goods Administration
TIDieR	Template for Intervention Description and Replication
UON	University of Newcastle
WHO	World Health Organization

1. Version control

The table below outlines the changes made to different versions of the protocol.

Version number	Overview of changes since previous version	Effective date
1.0	Nil. Inaugural protocol.	28/11/22
2.0	Updated based on feedback from HREC	24/1/23

2. Administrative information

2.1 Title

Identifying the effect of air purifiers in reducing the incidence of Acute Respiratory Infections (ARIs) among residents in Australian Residential Aged Care Facilities (RACFs): A Randomised Control Trial.

2.2 Trial registration

The trial will be registered with the Australia New Zealand Clinical Trial Registry (ANZCTR).

2.3 Funding, sponsors, and partners

This research is supported by an Australian Government Research Training Program (RTP) Scholarship.

The project is a clinical trial using air purifiers for identifying their effectiveness in reducing the incidence of ARIs in RACFs. Industry partner GAMA Health Care will sponsor air purifiers to support the study. They have no role in the study design, implementation, analyses, interpretation, or publications.

The project also requires statistical support. The guidance on design and statistical analysis will be arranged through Hunter Medical Research Institute (HMRI) Clinical Research Design & Statistics (CReDITSS) unit. The cost involving statistical support will be arranged by the principal investigator as part of the University of Newcastle's allocation of funding under the Research Training Package.

The project will be conducted in regional Residential Aged Care Facilities (RACFs) in NSW. The facility participation involves approval by a senior executive manager or delegate and/or the governing board. However, no cost is involved in the facility or participant enrolment process for the study.

3. Study team and governance

Study Team

Name	Role	Affiliations	Expertise	Responsibility and contributions
Associate Prof. Jenny Sim	Co-ordinating Principal Investigator/ Researcher	University of Newcastle	Student supervision<br>Quantitative research<br>Mixed methods<br>Patient safety<br>Nursing practice	Protocol review and updates<br>Governance<br>Student supervision
Bismi Thottiyil Sultanmuhammed Abdul Khadar	Principal Investigator	University of Newcastle	MSc. Nursing thesis on Clinical nursing leadership in preventing &controlling HCAIs in RACFs<br>Quantitative research	Literature Review<br>Study design<br>Protocol development<br>Project methodology<br>Ethics and governance
Prof. Brett Mitchell	Associate/ Assistant/ Sub- /Co- Investigator/ Researcher	University of Newcastle<br>Avondale University<br>Monash University	Clinical trial expertise<br>Quantitative research<br>Infection Prevention and Control	Research protocol<br>Student supervision
Dr. Julee McDonagh	Associate/ Assistant/ Sub- /Co- Investigator/ Researcher	University of Newcastle<br>University of Wollongong	Nursing practice<br>Quantitative research<br>Frailty and ageing research expertise	Research protocol<br>Student supervision

Governance

The project will be registered in the Research Ethics and Governance Information System (REGIS) as part of the ethics and its management. It has been assumed that the project will only involve negligible risk to any human participants which means there will be no foreseeable risk of harm or discomfort; and any foreseeable risk will be no more than an inconvenience (Buising et al., 2022; National Health and Medical Research Council (NHMRC) et al., 2018b).

The study will not involve any invasive procedures, but each participant will be exposed to the effect of an air purifier placed in the room. We do not anticipate any risks associated with the study. A minor inconvenience may be the noise of the air purifier; however, this can be negated by using a quiet function, which also stops any LED lights from being active.

Likewise, we do not foresee physical harm, psychological harm, exposure to illegal activity, economic harm, discrimination, devaluation, distress, or harm to the vulnerable population or any personnel involved in the trial.

4. Background

Australia's older population is growing at an exponential rate with the data reflecting 21% growth in older adults above 65 years of age by 2066 (Australian Institute of Health and Welfare (AIHW), 2021). Age related frailty and related immune senescence are known to be the leading factors responsible for older adults' susceptibility to various infections (Jaul & Barron, 2017; McElhaney et al., 2012; Wang & Casolaro, 2014). Childs et al. (2019) identified that the residents of RACFs are a highly vulnerable group of older adults and are more prone to get ARIs than any other population of older people. This is significant because ARIs are a major health concern among residents of RACFs (Childs et al., 2019).

While available data regarding potential routes of transmission is yet to provide clarity on the extent of transmission through various routes, live viruses and viral RNAs of common respiratory pathogens such as influenza and respiratory syncytial virus (RSV) have been identified in the samples of air collected from health care settings (Shiu et al., 2019). Although older adults in RACFs are susceptible to a variety of viral agents (Shi et al., 2020), influenza and RSV are known to be clinically important in this frail cohort (Branche & Falsey, 2015; Elliot & Fleming, 2008; Thompson et al., 2003; van Asten et al., 2012).

The transmission routes of various respiratory viruses have been identified as contact, droplet, and aerosols (Brankston et al., 2007; Leung et al., 2020; Tellier, 2006). However, the spread of viral agents through aerosols has received more attention over time as it has been noted as a substantial mode of transmission (Brankston et al., 2007; Leung et al., 2020; Tellier, 2006). Leung et al. (2020) identified that 35% of influenza viruses isolated were from the samples of aerosols and was comparably higher than that of the droplet samples. The airborne transmission can even occur over several metres of distance from the infected subject (Tellier et al., 2019).

Data from a recent study highlights the direct impact of poor indoor air quality in the

development of Severe Acute Respiratory Infections (SARI) and the role of advanced engineering measures in improving the quality of indoor air (Agarwal et al., 2021). This study also addresses the need for a combination of non-pharmacological and engineering measures including the utilization of air purifiers and modified ventilation systems for maintaining a robust indoor environment (Agarwal et al., 2021).

Further extended studies with distinctive research questions that investigate the benefits of the application of engineering measures for maintaining indoor air quality in RACFs have been warranted by Reddy et al. (2021). Also, trial-based projects to identify the pros and cons of employing filtration systems in RACF have been recommended (Reddy et al., 2021).

5. Methods

5.1 Aim

The study aims to evaluate the effectiveness of air purifiers in reducing the risk of acute respiratory infections (ARIs) among residents in Australian Residential Aged Care Facilities (RACFs).

5.2 Design

The proposed study will deploy a multi-centre double-blind randomised two-period two-treatment crossover design to determine the effectiveness of placing air purifiers in the rooms of the residents of RACFs. The overview of the crossover trial of the proposed project has been illustrated in figure.1.

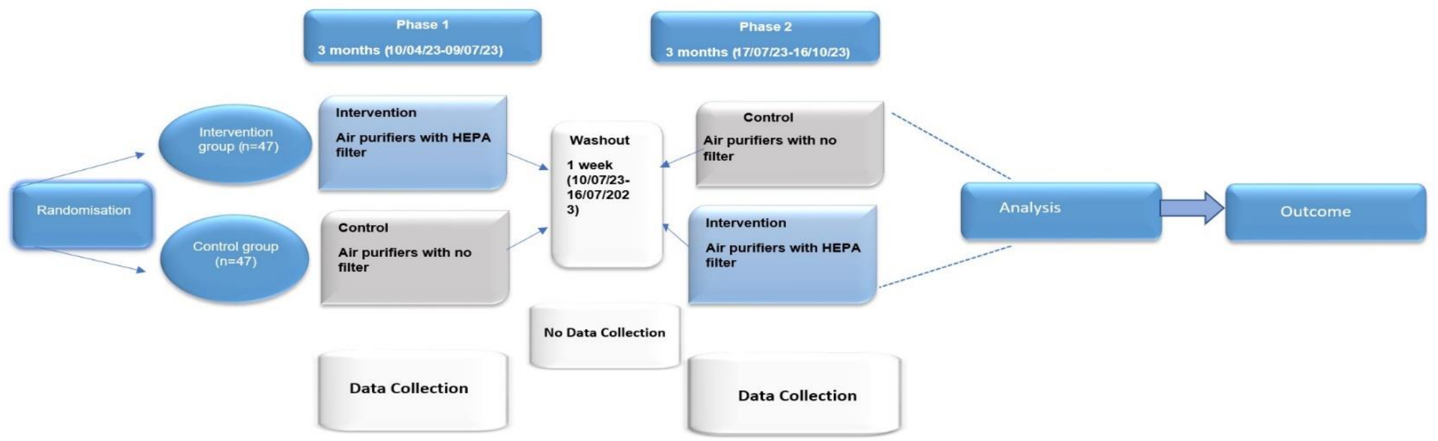

(Figure: 1. – study design).

5.3 Population and recruitment

Study setting

The proposed study will be a multi-centre trial. Two residential aged care facilities in a regional area of New South Wales will be invited to participate in the study. A purposive sampling approach based on the researcher's networks will be used to invite facilities.

To be eligible for participation, the residential aged care facilities must be accredited by the Aged Care Quality and Safety Commission (Australian Government Aged Care Quality and Safety Commission (ACQSC), 2022), be geographically based in New South Wales, Australia, and have a minimum of 50 residents.

Facility enrolment

To enrol in the study, the RACF will agree to these requirements:

1. in-kind support which would include facilitating access to the facility to enable recruitment including access to electronic and paper forms of potential participants' medical and health records, placement of air purifiers, and data collection.
2. nomination of one staff member as a local point of contact for staff, should any queries arise.
3. access to staff and/or communication to staff about the study and their support in keeping air purifiers turned on.
4. unless otherwise agreed to in writing, no use, disclosure, copying, or distribution of study materials or outcomes.

Participant recruitment

For potential participants who have the ability to provide informed consent, this will be sought. Mini-Mental State Examination (MMSE) (Folstein et al., 1975; Folstein et al., 1983) will be used to assess participants' ability to provide informed consent where required. For potential participants who lack the capacity to provide informed consent, a member of the research team will discuss the study with their representative, enduring power of attorney, or legal guardian (Information and Privacy Commission (IPC), 2021). In such circumstances, informed consent will be obtained from the relevant person with the authority to provide consent on behalf of the resident (Commonwealth Department of Health and Aged Care, 2015). The potential participants' guardians will be encouraged to discuss the research with the potential participant if the participants are able to understand what the study involves, based on their level of

cognitive impairment. Then it should be a joint discussion on whether the individual should participate in the study or not.

Participation in this study is entirely voluntary. This means that the participants do not need to agree to take part in the research, and participants' or their guardians' refusal to take part will also be respected. If any party decides to take part, they can still withdraw from the study at any time without having to give reasons for their choices/decisions. In such circumstances, any data collected concerning participants, or their guardians will be withdrawn from the study. Whatever the decision may be, the participants will be assured that it will not affect their treatment or relationship with the staff of the facility who are caring for the participants. Of the people caring for the participants, only the nursing staff and the members of the research team will be aware of participation or non-participation.

Potential participants who meet the eligibility criteria will be provided with information about the study. A member of the research team will follow up with potential participants to determine their interest in participating in the study. The follow-up period will be at least 3 days post-initial contact to allow participants time to consider participation.

Sometimes during the course of a study, new information becomes available about the treatment that is being studied and the participants will be kept informed of any significant new findings which may affect their willingness to continue in the study.

In circumstances where the potential participants require public guardians' approval since they are unable to provide consent, they will be excluded from the study.

In the enrolled facilities, a member of the research team will provide information to residents or their guardians about the study in paper forms. This will include the study requirement for accessing participants' electronic and paper forms of medical and health records managed by the enrolled facilities for obtaining information relevant to the study. This member of the research team will be available to answer any questions.

Participants' eligibility Criteria

Inclusion criteria

Participants must meet the following criteria to be included in the study. At the time of project commencement; the participants must:

- be a permanent resident of an Australian Residential Aged Care Facility enrolled in the study
- reside in their private room (i.e., not a shared room)

- not be on palliative Trajectory-C comfort care

Exclusion criteria

Potential participants may be excluded from the study if at the time of project commencement, they:

- are respite residents
- reside in shared rooms
- are on palliative Trajectory-C comfort care
- are unable to provide consent and require public guardian approval

5.4 Enrolment

Once ethics approval and governance approvals are in place, the facility will be officially enrolled. It is anticipated that governance approval will include approval by a senior executive manager or delegate and/or the governing board.

A participant will be considered enrolled in the study if they meet the eligibility criteria and once informed written consent has been obtained as necessary. Random allocation to the control or intervention arm of the study will only occur for enrolled participants.

Individual participants

The procedure of installing the air purifiers in participants' rooms will be mutually agreed upon between the research team and the potential participant or their nominated responsible person (enduring power of attorney or legal guardian). The final decision to participate in the study will be based on the best interest of the individual resident. Informed consent will be sought from each potential participant or their nominated responsible person (enduring power of attorney or legal guardian) before participating in the study. However, the potential participant or their nominated responsible person (enduring power of attorney or legal guardian) will have the option to withdraw from the study at any stage of the project. The participants will be randomly allocated into the intervention or control groups.

Delays

If there is a serious national epidemic during the trial, the study will continue to collect data, but any planned changeovers to the intervention will be delayed until it is possible to recommence. This is a practical move as the facility management and the staff will likely be diverted away from the research intervention in the case of a serious outbreak. An epidemic also has the potential to be a confounder of our primary and secondary outcomes, and so delaying the change from control to intervention will reduce the correlation between the epidemic and the intervention. If a participating facility fundamentally changes its case mix or similar impact as a result of an epidemic (e.g., influenza, RSV, or other ARI virus), the research team would decide

on how to handle this scenario based on the detail at the time. Options would include excluding data collected from that facility from analysis and/or stopping data collection from the facility. Any decision from the research team would need to consider risk (personal, residents, community), public health and government requirements or directives, and impact on the quality of the study.

Discontinuation of study or study site

The study will be discontinued if a regulatory body, funding body, or Human Research Ethics Committee (HREC) judges it necessary for medical, safety, regulatory, or other reasons consistent with applicable laws, regulations, and good clinical practice.

5.5 Intervention

The intervention process described fulfills the requirements suggested in the TIDieR checklist (Hoffmann et al., 2014). The TIDieR checklist involves a checklist and guide known as Template for Intervention Description and Replication (TIDieR) developed by an international group of experts and stakeholders to improve the completeness of reporting and ultimately the replicability of interventions (Hoffmann et al., 2014). All participants will receive an air purifier which may contain a High-Efficiency Particulate Absorbing (HEPA) filter for air purification (intervention) or not contain a HEPA filter (control).

The guideline for placing air purifiers in healthcare facilities for their effective functioning has been proposed by the Department of Health Government of Western Australia (2022). The air purifier will be placed in the room of each participating resident and positioned in the closest suitable proximity to the resident (point of source). There should be a minimum distance of ten centimetres between the equipment and the wall to ensure air circulation. To ensure free air flow of the air purifier, the air intake and outlet of the air purifier should be kept unhindered. There should be a clear space of at least 25 cm to the front of the air purifier. The air purifier will be positioned away from any heating or cooling sources, and it will be placed on a stable and solid surface.

The placement of air purifiers will be undertaken by one of the members of the research team who is responsible for collecting or determining outcome data. Communal and shared areas such as recreation rooms, lounges, dining areas, and double rooms will not contain an air purifier, as the study is seeking to determine the effect of air purifiers in an individual resident’s room.

The first phase of the study will continue for three months. After the first phase of the study, a washout period of one week will be applied. Since the air purifier function is based on instant air

purification with the aid of HEPA filters, the chances of carryover effects are expected to be quite unlikely. Also, a long washout period may lead to missing data in the epidemiological peak of the trial phases, which may in turn further influence the outcome analysis. However, this period will be required to swap / change air purifiers between groups and for identifying and eliminating carryover effects, if any.

During the washout period, residents in the control phase will have their air purifier changed or modified and subsequently have an air purifier with HEPA filtration. Conversely, for residents who completed an intervention, they will have HEPA filtration removed in preparation for the control phase.

The person responsible for changing HEPA filtration will be a member of the research team who is not responsible for collecting or determining outcome data. The changeover process will occur in a way in which the resident remains blinded to the intervention. For example, a HEPA filter will not be removed or installed in view of a resident. These practices will ensure the double-blinding of the trial.

When air purifiers are moved between participants, they will be cleaned by a member of the research team in accordance with the manufacturer’s instructions. External surfaces of the air purifier will be cleaned with a Therapeutic Goods Administration (TGA) registered disinfectant wipe with claims of activity against viruses and bacteria. There is no airborne transmission infection risk to residents with regards to moving air purifiers between residents (if this was required to be done).

The second phase of the study will continue for another three months.

Fidelity

The fidelity of the trial will be ensured by arranging random checks or observations made by the selection of nominated facility staff on the air purifiers and participants of the trial. These staff will have received training on the operation of air purifiers. They will check to identify if the air purifiers are turned on and to make sure that they are in active mode for their effective functioning. Minimum monthly checks like this will be carried out by the facility staff. In circumstances where an alert on the air purifier is identified, the member of the research team who is involved in the data collection will be notified immediately.

Regular checks on participants will also be done by the nominated staff member of the facility to estimate the time that participants spend outside the room to recognise the effectiveness of the intervention. This is important as it influences participants’ exposure to the intervention and consequently, the outcome. For example, the air purifiers will be placed in the resident’s room

and the duration of residents' exposure to air purifiers is a significant part of the study that may impact the outcome of the intervention. Any breakout period where there is a long gap in participants' exposure to air purifiers (such as spending outside the room for long hours, a day outing, days of leave, and hospitalisation) will be recorded by the nominated staff. This will be collected and recorded carefully by the member of the research team who is involved in data collection for it to be included in the outcome analysis for identifying the effectiveness of the intervention.

5.6 Outcomes

Primary outcome

The primary outcome is a reduction in the occurrence of ARIs. Each participant will have a binary outcome, no infection, and infection, for their control and intervention study period. The case definition for Acute Respiratory Infection (ARI) is the sudden/acute onset of at least one of the following four respiratory symptoms:

- cough
- sore throat
- shortness of breath
- coryza

AND

- a clinician's judgement that the illness is due to an infection (European Centre for Disease Prevention and Control (ECDC), 2018). This definition is consistent with World Health Organization (WHO) Regional Office for Europe (2011).

An ARI will then be categorised as one of the following:

- ARI, no diagnostic test performed: Residents presenting with symptoms as identified by the definition of ARI and eligible to be categorized as an ARI case.
- ARI, influenza (A and B): Residents presenting with symptoms of ARI and confirmed influenza (A and B) by diagnostic lab tests.
- ARI, SARS Cov2: Residents presenting with symptoms of ARI and confirmed SARS Cov2 by institutional RAT or diagnostic lab tests.
- ARI, rhinovirus: Residents presenting with symptoms of ARI and confirmed rhinovirus by diagnostic lab tests.

- ARI, respiratory syncytial virus: Residents presented with symptoms of ARI and confirmed RSV by diagnostic lab tests.
- ARI, adenovirus: Residents presenting with symptoms of ARI and confirmed adenovirus by diagnostic lab tests.
- ARI, diagnostic test performed but no virus identified

Participants in the study will not be subject to a diagnostic test as part of the study. Any diagnostic tests will be determined as part of their medical care, facility procedure, or usual practice. We expect any ascertainment bias to remain constant throughout the study i.e., the same in both phases.

Secondary outcomes

The secondary outcomes of this study are:

- Time to first infection for participants. This will be defined as time in days since the commencement of the study phase to the diagnosis of ARI.
- The number of visits to an emergency department due to an ARI
- The number of hospital admission due to an ARI. A hospital admission is defined as being admitted to a hospital and having at least one overnight stay.
- The number of medical consultations including GP and NP reviews due to an ARI. A consultation will be defined as a billable visit to a medical or nurse practitioner and/or where a medical or nurse practitioner has documented an assessment related to an ARI in the participants' medical notes at the facility.

5.7 Sample size

An estimate of a 50% reduction in ARI by keeping 40% as the current incidence rate requires a 40% respiratory infection proportion in the control group and a 20% proportion in the treatment group. For the AB-BA sequence of the crossover trial, the required number of participants will be 94 in total i.e., 47 for the AB sequence and another 47 for the BA sequence.

Assuming the proportion of respiratory infections under control conditions is 40% and the expected proportion of respiratory infections is 20% when using air purification, a cross-over trial with 94 participants (47 in each sequence) will have 80% power to detect this difference at the 5% significance level. This corresponds to an odds ratio of 0.375 for air purification (filtration) vs control (no filtration). The calculation of sample size has been illustrated in figure.2.

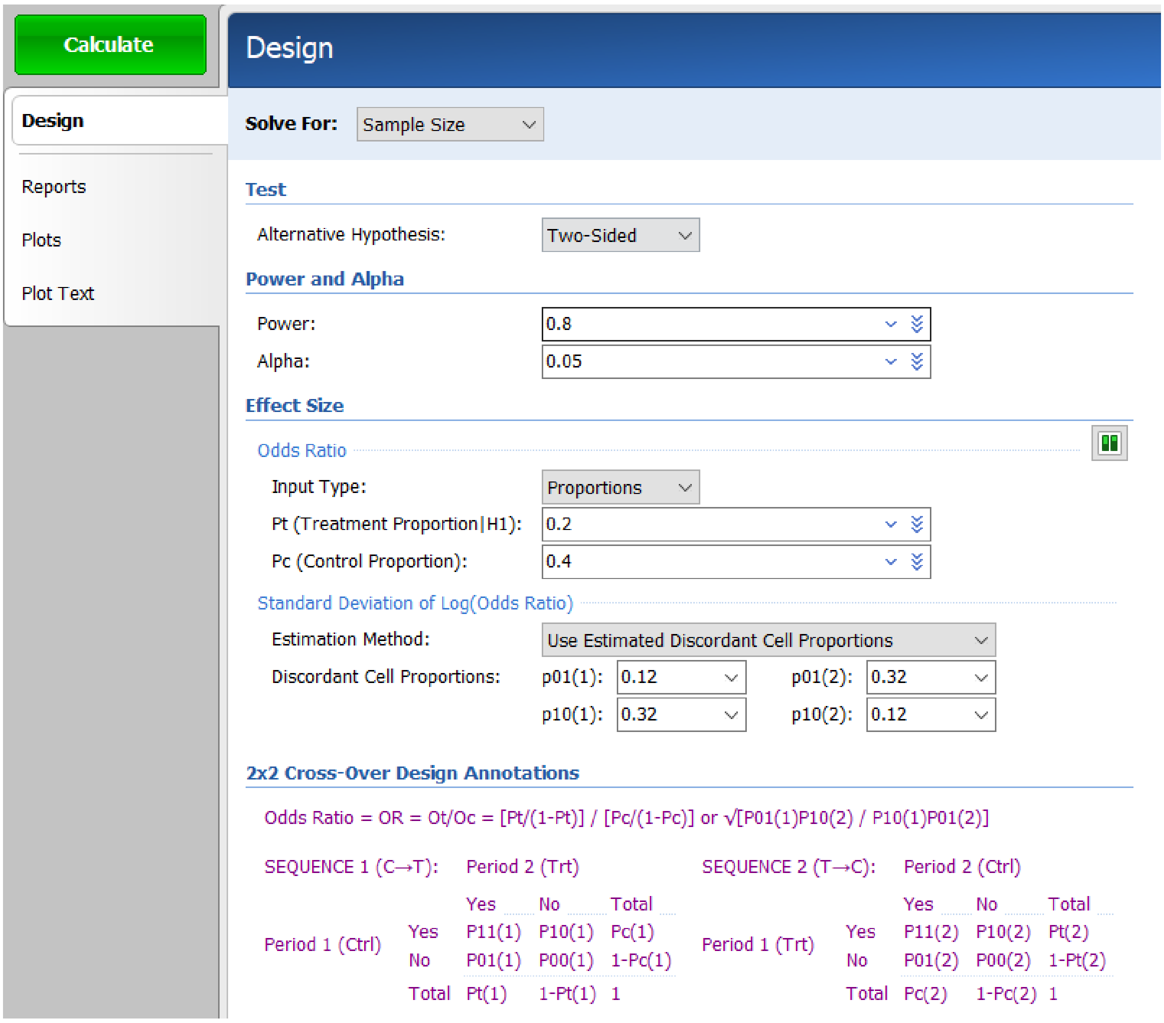

Figure: 2. Sample size calculation of the proposed project

The assumptions for the proportions of participants with a respiratory infection are based on data from a systematic review (Childs et al., 2019). It is now widely accepted that respiratory viruses are transmitted via aerosols and that transmission via inanimate surfaces is not very common and for some respiratory pathogens, such as SARS-COV2, potentially negligible (Greenhalgh et al., 2021; Tang et al., 2021). Therefore, if a person has clean air the likelihood of transmission is negligible and improving air quality is likely to have a significant role in reducing risk of infection. There have been several studies that support the use of air purification as a way to reduce the risk of respiratory infections.

- In a COVID-19 ward, 99% of aerosols were cleaned within 5 minutes, by use of an air purifier (Buising et al., 2022).
- Simulation studies, including one on a classroom where the use of an air purifier was found to have 70-90% reduction in transient aerosols (Burgmann & Janoske, 2021)

Given the topic, there have been no RCTs or quasi-experimental studies that have examined reductions in patient outcomes (respiratory infection) when using air purifiers. In part, this is why

this study is critical. With the above justification and context in mind, we have also relied on expert opinion when determining the 50% reduction figure. These have included those from a range of disciplines – infectious disease, infectious control and aerosol science. Expert opinion includes people who have advised national government on Australia’s pandemic response. Please also note that in our sample size estimate, we have also been conservative with the baseline incidence rate, which is likely to be higher.

5.8 Randomisation and concealment

Prior to the enrolment of participants, all air purifiers in the study will be pre-labelled. Air purifiers will either have a HEPA filter removed (control) or will have the air purifier remain in place (intervention). A database listing the air purifier (label) and whether it is a control or intervention will be established. This database will be held by one researcher (researcher 1) not involved in the enrolment (including recruitment and consent), data collection, or decisions regarding the outcome. Other members of the research team will enrol participants. When a participant is enrolled, the researcher will notify researcher 1 of their name. No other baseline or demographic data will be provided.

Researcher 1 will randomise participants using a protocol of block randomisation stratified by site. This approach will ensure and even balance of participants in the intervention and control arms at each site. Randomisation will occur in a 1:1 ratio into one of the two sequences of intervention and control groups using a computer-generated randomisation schedule. Allocation concealment will be ensured as the same researcher will not disclose the outcome of randomisation to the remainder of the team and will hold the allocation in a password-protected database on a file not accessible to the remainder of the team. Upon the immediate commencement of the trial at a site and after all baseline data is collected, researcher 1 will inform the remainder of the team of which air purifier should be provided to which participant. As air purifiers are already pre-labelled, researchers allocating the air purifiers to participants will not be able to visually tell whether an air purifier has a filter (intervention) or not (control).

5.9 Blinding

The study is double-blinded. The participant will be unaware of whether their air purifier contains a HEPA filter or not. The researcher determining the outcome (whether a participant has an ARI) will also be unaware of whether the participant has an air purifier with or without a HEPA filter. The procedure of changing the air purifiers will not involve any disclosure to participants or the researcher collecting outcome data about whether the air purifier has a HEPA filter. The

unblinding of the participant's allocation to a treatment group will only occur immediately prior to the final analysis being undertaken.

5.10 Data collection

The duration of the study is 6 months. The intended starting date for the study is April 2023. This will allow the change between phases to coincide with the peak period of the influenza season, thus allowing a relatively equal distribution of potential influenza risk in both phases, based on historical epidemiological data**.** After enrolment in the study, a researcher not involved in the randomisation process will be responsible for collecting **baseline and demographic data**.

This **baseline and demographic data** will include:

- Age.
- Sex.
- Past medical history.
- History of an ARI (if any) with its diagnosis and management including hospitalization.
- Details on any respiratory infection
- Current medications including cytotoxic, antibiotics, and antivirals.
- Vaccination status.
- Current Advanced Care Directive.
- Facility residency status.
- Confounders including
    - Mobility status (eg. Independent, Standby Assist, Assist x 1, Assist x 2, Immobile)
    - Resident's room size
    - Resident's room heating, ventilation and air conditioning (HVAC) system.

The processes for collecting this baseline and demographic data are presented in the table below.

**Data Item**	**Source of data**	**Data Extraction**	**Person Responsible**
Age	Demographic details from resident record	Manual data entry onto online form using iPad	Ms Bismi Thottiyil Sultanmuhammed Abdul Khadar (BTSAK)
Sex	Demographic details from resident record	Manual data entry onto online form using iPad	BTSAK
Past medical history	Data collated from:<br>▪ Review of residents' medical record<br>▪ Review of National Screening and Assessment Forms (NSAFs)<br>▪ Review of Aged Care Facility clinical record	Manual data entry onto online form using iPad	BTSAK

	▪ Verbal report of participants/legal guardian		
History of an ARI (if any) with its diagnosis and management including hospitalization	Data collated from:<br>▪ Review of residents' medical record<br>▪ Review of Patient Health summary from GP<br>▪ Review of Hospital Discharge Summary (if any)<br>▪ Review of Aged Care Facility nursing records<br>▪ Verbal report of participants/legal guardian	Manual data entry onto online form using iPad	BTSAK
Details on any respiratory infection	Data collated from:<br>▪ Review of residents' medical record<br>▪ Review of Patient Health summary from GP<br>▪ Review of Hospital Discharge Summary (if any)<br>▪ Review of Aged Care Facility nursing records<br>▪ Verbal report of participants/legal guardian	Manual data entry onto online form using iPad	BTSAK
Current medications including cytotoxic, antibiotics, and antivirals	Data collated from:<br>▪ Review of current patient health summary from GP<br>▪ Review of current medication chart<br>▪ Review of Hospital Discharge Summary (if any)	Manual data entry onto online form using iPad	BTSAK
Vaccination status (including type and dates of current vaccinations)	Data collated from:<br>▪ Review of Aged care facility records<br>▪ Review of resident's medical record<br>▪ Contacting GP practice for updates (if required)	Manual data entry onto online form using iPad	BTSAK
Current Advanced Care Directive.	Data collated from:<br>▪ Review of Aged care facility records<br>▪ Discussion with participants/legal guardians	Manual data entry onto online form using iPad	BTSAK
Facility residency status	Data collated from:<br>▪ Review of Aged care facility records	Manual data entry onto online form using iPad	BTSAK
Mobility status (eg. Independent, Standby Assist, Assist x 1, Assist x 2, Immobile)	Data collated from:<br>▪ Review of Aged care facility electronic clinical nursing records<br>▪ Resident current mobility assessments and care plans kept by the aged care facility<br>▪ Verbal report from registered nurses<br>▪ Review of Physiotherapist's assessments<br>▪ Verbal reports from physiotherapists	Manual data entry onto online form using iPad	BTSAK
Resident's room size	Data provided by aged care facility manager	Manual data entry onto online form using iPad	BTSAK
Resident's room heating, ventilation and air conditioning (HVAC) system	Data provided by aged care facility manager	Manual data entry onto online form using iPad	BTSAK

During the course of the study, a researcher will visit participating facilities fortnightly to collect

routine data. During this visit, the researcher will screen the medical, nursing, and laboratory records of all participants for any evidence of an ARI. Where an ARI is determined, the following data will be collected:

- Signs and symptoms of ARI with the date of onset of the first symptom.
- Laboratory tests (if any)
  - Rapid Antigen Test (RAT) results (if any).
  - RT-PCR/culture/Serum Examination (SE) result (if any).
- The number of medical consultations including General and Nurse Practitioners' reviews due to an ARI.
- The number of visits to an emergency department due to an ARI.
- The number of hospital admissions due to an ARI.
- Confounders will also be captured during routine data collection each fortnight and will include
  - the resident's documented mobility status,
  - changes to current medications (including cytotoxic, antibiotics, and antivirals)
  - any additional vaccinations received.

The processes for collecting this routine data are presented in the table below.

**Data Item**	**Source of data**	**Data Extraction**	**Person Responsible**
Signs and symptoms of ARI with the date of onset of the first symptom.<br>▪ cough<br>▪ sore throat<br>▪ shortness of breath<br>▪ coryza<br>AND<br>▪ clinician's judgement that illness is due to an infection	Data collated from:<br>▪ Review of residents' medical record<br>▪ Review of Aged Care Facility clinical record<br>▪ Review of Patient Health summary from GP<br>▪ Review of Aged Care Facility nursing records<br>▪ Verbal report from registered nurses<br>▪ Verbal report of participants/legal guardian	Manual data entry onto online form using iPad	Ms Bismi Thottiyil Sultanmuhammed Abdul Khadar (BTSAK)
RAT performed (Y/N)	Data collated from:<br>▪ Review of residents' medical record<br>▪ Review of Aged Care Facility clinical record<br>▪ Review of Patient Health summary from GP<br>▪ Review of Aged Care Facility nursing records	Manual data entry onto online form using iPad	BTSAK
Rapid Antigen Test (RAT) results (if any).	Data collated from:<br>▪ Review of residents' medical record<br>▪ Review of Patient Health summary from GP<br>▪ Review of Hospital Discharge Summary (if any)	Manual data entry onto online form using iPad	BTSAK

	▪ Review of Aged Care Facility nursing records<br>▪ Laboratory records		
Swab (RT-PCR/culture/SE) sent (Y/N)	Data collated from:<br>▪ Review of residents' medical record<br>▪ Review of Aged Care Facility clinical record<br>▪ Review of Patient Health summary from GP<br>▪ Review of Aged Care Facility nursing records	Manual data entry onto online form using iPad	BTSAK
RT-PCR/culture/Serum Examination (SE) result (if any).	Data collated from:<br>▪ Review of residents' medical record<br>▪ Review of Patient Health summary from GP<br>▪ Review of Hospital Discharge Summary (if any)<br>▪ Review of Aged Care Facility nursing records<br>▪ Laboratory records	Manual data entry onto online form using iPad	BTSAK
Medical consultations including General Practitioner and/or Nurse Practitioners' reviews due to an ARI (recording date of each consultation)	Data collated from:<br>▪ Review of residents' medical record<br>▪ Review of Patient Health summary from GP<br>▪ Review of Aged Care Facility nursing records	Manual data entry onto online form using iPad	BTSAK
Treatment commenced (Y/N, specify)	Data collated from:<br>▪ Review of residents' medical record<br>▪ Review of Patient Health summary from GP<br>▪ Review of Hospital Discharge Summary (if any)<br>▪ Review of Aged Care Facility nursing records	Manual data entry onto online form using iPad	BTSAK
The number of visits to an emergency department due to an ARI (recording date of each visit)	Data collated from:<br>▪ Review of residents' medical record<br>▪ Review of Patient Health summary from GP<br>▪ Review of Hospital Discharge Summary (if any)<br>▪ Review of Aged Care Facility nursing records	Manual data entry onto online form using iPad	BTSAK
The number of hospital admissions due to an ARI (recording dates and length of stay of each admission)	Data collated from:<br>▪ Review of residents' medical record<br>▪ Review of Patient Health summary from GP<br>▪ Review of Hospital Discharge Summary (if any)<br>▪ Review of Aged Care Facility nursing records	Manual data entry onto online form using iPad	BTSAK
The resident's documented mobility status,	Data collated from:<br>▪ Review of Aged care facility electronic clinical nursing records<br>▪ Resident current mobility assessments and care plans kept by the aged care facility<br>▪ Verbal report from registered	Manual data entry onto online form using iPad	BTSAK

	nurses<br>▪ Review of Physiotherapist's assessments<br>▪ Verbal reports from physiotherapists		
Changes to current medications (including cytotoxic, antibiotics, and antivirals)	Data collated from:<br>▪ Review of current patient health summary from GP<br>▪ Review of current medication chart<br>▪ Review of Hospital Discharge Summary (if any)	Manual data entry onto online form using iPad	BTSAK
Update of any additional vaccinations received.	Data collated from:<br>▪ Review of Aged care facility records<br>▪ Review of resident's medical record<br>▪ Contacting GP practice for updates (if required)	Manual data entry onto online form using iPad	BTSAK

To capture each resident's 'typical' pattern of mobility, a member of the research team will interview either the resident or a staff member who is familiar with the resident to document their daily routine. This **mobility data** will include time spent in the dining room, activities room or in other social activities and aims to capture a baseline of time spent out of their room as part of regular or routine activities. This will then be quantified into an estimate of hours spent outside the room on a 'typical' day for the purpose of assessing confounders. Data on the pattern of mobility will be collected in Phase 1 and Phase 2 of the trial to ensure that changes over time are captured.

Onsite data collection in participating facilities will be carried out by a member of the research team (Ms Bismi Thottiyil Sultanmuhammed Abdul Khadar) who is blinded to the intervention. Data will include **baseline and demographic data** and **routine data** (collected each fortnight) and **mobility data** collected within each phase of the project. This data will be collected and recorded electronically via an iPad using an online data collection form created in QuestionPro which is the University of Newcastle approved software for data collection and will be exported as an Excel spreadsheet. The data will be downloaded from QuestionPro and stored in the University of Newcastle supported storage solution called SharePoint, a cloud-based document management system, which facilitates sharing of information between the members of the team. The collected data will be safely and securely stored in accordance with the Australian Code for the Responsible Conduct of Research, 2018 (National Health and Medical Research Council (NHMRC) et al., 2019), and as per University policy and procedure on management of research data (University of Newcastle (UON), 2017). The data collected will be stored in a password-protected (two-factor authentication) electronic database, on a University of Newcastle server. The drive folder can only be accessed by research team members with password

protection. The data collection instrument’s access is limited to only the people who have permission to review that information.

The data will be managed as per NHMRC guidelines of responsible conduct of research including within its scope the appropriate generation, collection, access, use, analysis, disclosure, storage, retention, disposal, sharing, and re-use of data and information (National Health and Medical Research Council (NHMRC) et al., 2019). The safe retention and use of research data for maintaining research integrity will be ensured by adhering to the codes; the Australian Code for the Responsible Conduct of Research 2018 (NHMRC et al, 2018a) and the National Statement on Ethical Conduct in Human Research (updated 2018) (NHMRC et al., 2018b). The management including storage of data will be undertaken in accordance with UON policies of the responsible conduct of research which includes the storage, retention, disposal, and re-use of data and primary materials to ensure justification of potential outcomes of the research for strengthening the value of the research data for future research (The University of Newcastle (UON), 2017). The ethical protocols of HREC (UON) in relation to privacy, confidentiality, and data protection will be abided by.

5.11 Data analysis

Descriptive statistics of the sample will report demographics and clinical characteristics stratified by treatment order. An alpha threshold of 0.05 will be used to assess statistical significance and the reported results will include 95 % confidence intervals. Due to the high likelihood of participants being lost-to-follow-up in this sample, demographics and clinical characteristics will be used to conduct the analysis using multiple imputations.

Primary outcome

The effectiveness of treatment will be assessed by comparing the proportion of infections over the two cross-over study periods. Each participant will have a binary outcome, no infection, and infection, for their control and intervention study period and analysis will include fixed effect for each site. The treatment effect will be measured using logistic regression mixed effects models, where the main effects in the model will be treatment, study collection time, and cross over design characteristics. A random intercept effect for an individual will correct for non-independent observations made between the cross over study periods. The model will estimate an odds ratio that will compare the odds of infection when having air filtration active against not having air filtration active.

As the mean incubation and infectious period for common respiratory viral pathogens is less than 7 days, a 1-week washout period will be sufficient within this study. Where a person

acquires an infection during a phase, the infection will be attributed to that phase (noting that we are measuring infection as a dichotomous variable). Because our measurement of infection is a dichotomous variable, symptoms must resolve before a new infection can be counted.

Secondary outcome

Time to respiratory infection will be modelled using survival analysis with lost-to-follow-up and death to be considered as censoring events. Kaplan-Meir curves will be used to estimate the survival function. A mixed-effect cox regression model will be used to assess the change in hazards between the treatment and control arms of the study, where a random effect will account for within patient correlation. The effect of the treatment will be assessed by the hazard ratio, which will compare the change in hazards between the two-treatment cross-over arms. Assuming the proportion of respiratory infections under control conditions is 40% and the expected proportion of respiratory infections is 20% when using air purification, a cross-over trial with 94 participants (47 in each sequence) will have 80% power to detect this difference at the 5% significance level. This corresponds to an odds ratio of 0.375 for air purification vs control.

5.12 Confounders

The proposed project will identify the effect of air purifiers in reducing the ARIs in RACFs through aerosol filtration. The study will only measure the activities and aerosol exposure in the rooms of residents in enrolled facilities. The potential confounder may be the resident's exposure to aerosols outside the rooms such as communal and shared areas including recreation rooms, lounges, dining areas, and double rooms since these will not contain air purifiers. Each resident's mobility status will be captured as part of baseline demographic data and in routine data collection each 2 weeks to document changes in mobility patterns over the course of the study. In addition, a cross-sectional audit of each resident's movement on a 'typical' day will be captured during each phase of the study. This data will be quantified to estimate the average amount of time a resident spends outside their room each day in hours. Any treatment for an ARI using antiviral or antibiotic may also be considered as another confounder. Hence, any participants who are on treatment for an ARI throughout the trial period will also have this treatment monitored and documented accordingly. Routine data collection will include changes to current medications (including cytotoxic, antibiotics, and antivirals) to ensure this potential confounder is captured and quantified.

An epidemic also has the potential to be a confounder of primary and secondary outcomes.

6. Monitoring

The member of the research team blinded to the intervention will be responsible for fortnightly data collection. The data collection and the trial processes will be monitored by the members of

the research team who are the supervisors of the project. Data will be reviewed to ensure the correct collection of the data sets and implementation of the intervention. Support (site visits, telephone, and email contact) will be provided by the study staff as required during the intervention phase to check core trial processes and maintain data quality.

6.1 Safety evaluations

It has been assumed that the project will only involve negligible risk to any human participants which means there will be no foreseeable risk of harm or discomfort; and any foreseeable risk will be no more than an inconvenience (National Health and Medical Research Council (NHMRC) et al., 2018b). The study will not involve any invasive procedures, but each participant will be exposed to the effect of an air purifier placed in the room. We do not anticipate any risks associated with the study. A minor inconvenience may be the noise of the air purifier; however, this can be negated by using a quiet function, which also stops any LED lights from being active.

Staff training, on the functioning of the air purifier, will be arranged for the participating facilities. Staff will be registered or enrolled nurses in the aged care facilities who will have experience in managing individuals with cognitive impairment and people with diagnoses of anxiety or depression. Participants who show any signs of distress will be commenced on the behaviour chart, consistent with local practice and local policy.

The principal investigator will attend the facilities on a fortnightly basis and as part of the data collection process. In circumstances where there is an identification that the air purifier is causing distress, the situation can be managed immediately by using a quiet function, which also stops any LED lights from being active.

If the air purifier continues to cause distress, the air purifier can be removed from the participant's room and the participant will be withdrawn from the study.

Likewise, we do not foresee physical harm, psychological harm, exposure to illegal activity, economic harm, discrimination, devaluation, distress, or harm to the vulnerable population or any personnel involved in the trial. However, continuous follow-up and safety evaluation checks will be carried out throughout the trial period. This will be done as part of the data collection process and by maintaining constant communication with the nominated staff of the participating facilities.

6.2 Adverse event reporting

No adverse events are anticipated from the study. In circumstances where any inconvenience

or adverse events are observed or reported by the facility, they will be collected and recorded in the source documents by the principal supervisor. The principal supervisor should notify the approving ethics committee of serious adverse events occurring at any of the sites. Adverse events could require reporting as per facility specific policy.

6.3 Incident monitoring and reporting

The principal supervisor is responsible for ensuring that all incidents observed by the investigator or reported by sites are collected, reviewed, and recorded in the source documents. Incidents could require reporting as per facility specific policy and/or notification to the approving ethics committee.

7. Consent

7.1. Overview

In the enrolled facilities, a member of the research team will provide information to residents about the study in paper form and be available to answer any questions. For potential participants who have the ability to provide informed consent, this will be sought. Also, participants' medical records and National Screening and Assessment Forms (NSAFs) will be reviewed for the diagnoses of dementia and/or other mental illnesses such as depression and anxiety disorders.

Participants' degree of cognitive impairment will be assessed by administering the Mini-Mental State Examination (MMSE) which is a 30-point tool for assessing cognitive impairment and recommends a score of 24 as an indication of normal functioning. Any score of 23 or below indicates dementia.

Mini-Mental State Examination (MMSE) (Folstein et al., 1975; Folstein et al., 1983) will be used to assess participants' ability to provide informed consent where required. For potential participants who lack the capacity to provide informed consent, a member of the research team will discuss the study with their representative, enduring power of attorney or legal guardian (Information and Privacy Commission (IPC), 2021). In such circumstances, informed consent will be obtained from the relevant person with the authority to provide consent on behalf of the resident. In circumstances where the potential participants are unable to provide consent and require public guardian approval, they will be excluded from the study.

Potential participants who meet the eligibility criteria or their guardians will be provided with information about the study. A member of the research team will follow up with potential participants or their guardians, to determine their interest in participating in the study. The follow-up period will be at least 3 days post-initial contact, to allow potential participants or

their guardian time to consider participation. The potential participants and guardians will be informed of the risks and benefits of participating in the trial. The risks may include a minor inconvenience such as the noise of the air purifier; however, this can be negated by using a quiet function, which also stops any LED lights from being active. Likewise, the research team does not foresee physical harm, psychological harm, exposure to illegal activity, economic harm, discrimination, devaluation, distress, or harm to the vulnerable population or any personnel involved in the trial.

The participants or their guardians will also be informed that the potential benefits of this trial are reducing the risk of acute respiratory infection for participants. The broader benefits of this research are informing the value of improving air quality in residential aged care facilities more broadly, potentially informing future policy and/or guidance. There will not be any financial benefits or disadvantages to the members of the research team or participants of the project

The participant information form explaining the details will be given to potential participants or their guardians.

As part of the consenting process, the participants or guardians will also be informed that the air purifiers placed in the participants' room will be available for their continued use followed by the completion of a clinical trial. There are no conditions attached to participants' continued use of the air purifier, however, the cost of changing air filters after the study has been completed will be at the expense of the participants. This will also be detailed in the participant information form. If they do not wish to keep the air purifier, it will be removed by the research team.

8. Data Management

8.1 Privacy and data management

The project is supervised and overseen by a clinician, registered with the Australian Health Practitioner Regulation Authority. The data collection will also be carried out by an RN registered with the Australian Health Practitioner Regulation Authority (AHPRA). The collected data will be safely and securely stored in accordance with the Australian Code for the Responsible Conduct of Research, 2018 (National Health and Medical Research Council (NHMRC) et al., 2018a) and as per university policy and procedure on management of research data (University of Newcastle (UON), 2017). As part of the data collection process, the individual resident details will be linked to a QR code. Once the resident details (QR codes) are removed, shortly after data collection, no re-identifiable information will be held or stored. All participant information will be anonymous.

In circumstances where any participants or their guardians decide to withdraw their participation from the study, all the information collected from and about them will be withdrawn from the

study data and will be destroyed. But in some cases, the data would have already been included in the study database or included in the analysis which may not be withdrawn. The participant or their guardian will be notified of keeping and including already entered data in the final analysis. This data will be kept confidential and will be destroyed in accordance with Commonwealth Privacy Laws and the NSW Health Records and Information Privacy Act 2002.

All the information collected from the participants or their guardians for the study will be treated confidentially, and only the members of the research team and staff providing care for participants in the residential aged care facility will have access to it. The study results may be presented at a conference or in a scientific publication, but individual participants will not be identifiable in such a presentation. The participant's personal information will be accessed, used, and stored in accordance with Commonwealth Privacy Laws and the NSW Health Records and Information Privacy Act 2002.

8.2 Confidentiality and data management

The confidentiality of the participants involved in the trial will be maintained by appropriately storing the data in password-protected files and ensuring its destruction after the completion of the study. The collected data will be stored in a password-protected (two-factor authentication) electronic database, on a University of Newcastle server. The data will be kept for a period of 15 years from the point of any publication relating to the research.

After enrolment in the study, one researcher, not involved in the randomisation process, will be responsible for collecting baseline and demographic data. An onsite data collection by visiting participating facilities will be carried out by a member of the research team who gets blinded to the intervention. This data will be collected and recorded electronically via an iPad using an online form created in QuestionPro which is the University of Newcastle approved software for data collection and will be entered into an electronic database called Excel spreadsheet. The data will be downloaded from QuestionPro and stored in the University of Newcastle supported storage solution called SharePoint, a cloud-based document management system, which facilitates sharing of information between the members of the team. The collected data will be safely and securely stored in accordance with the Australian Code for the Responsible Conduct of Research, 2018 (National Health and Medical Research Council (NHMRC) et al., 2019), and as per University policy and procedure on the management of research data (University of Newcastle (UON), 2017). The data collected will be stored in a password-protected (two-factor authentication) electronic database, on a University of Newcastle server. The drive folder can only be accessed by research team members, with password protection. The data collection instrument's access is limited to only the people who have permission to review that

information.

The data will be managed as per NHMRC guidelines of responsible conduct of research including within its scope the appropriate generation, collection, access, use, analysis, disclosure, storage, retention, disposal, sharing, and re-use of data and information(National Health and Medical Research Council (NHMRC) et al., 2019). The safe retention and use of research data for maintaining research integrity will be ensured by adhering to the codes; the Australian Code for the Responsible Conduct of Research 2018 (NHMRC et al, 2018a) and the National Statement on Ethical Conduct in Human Research (updated 2018) (NHMRC et al., 2018b). The management including storage of data will be undertaken in accordance with UON policies of the responsible conduct of research which includes the storage, retention, disposal, and re-use of data and primary materials to ensure justification of potential outcomes of the research for strengthening the value of the research data for future research (The University of Newcastle (UON), 2017). The ethical protocols of HREC (UON) in relation to privacy, confidentiality, and data protection will be abided by.

8.3 A plan for making information arising from the research available

The participating facility may be interested in using the data obtained from this study to inform local priorities and practices. Specifically, this may contain facility-based infection data. This data would be provided subject to any other ethical considerations. This data would still be aggregated and not at the participant level. The research team will comply with the open access policy of the NHMRC.

8.4 Possibility of commercial exploitation

The data will be collected subsequently following the commencement of the intervention or placement of air purifiers in rooms of participants of enrolled RACFs. The collected data will only be used to identify the symptoms of ARIs in participants or any new ARI cases followed by the commencement of the intervention. There will not be any financial benefits or disadvantages to the members of the research team or participants of the project.

9. Human Research Ethics

9.1 Overview

A copy of the protocol, other written participant information including, and any other relevant study material will be submitted to the HREC for written approval. The principal investigator must submit and, where necessary, obtain approval from each UON and Hunter New England (HNE) HRECs for all subsequent protocol amendments, once approved by the management committee, and changes to the informed consent document.

The principal investigator should notify the HREC of deviations from the protocol or serious adverse events occurring at the enrolled RACFs in accordance with local procedures. The investigators will be responsible for adhering to ethics committee requirements throughout the study.

9.2 Confidentiality

The facility identifiers will be assigned. All data and information generated as part of the study will be kept confidential by members of the research team. The research team or other facility staff will not use this information and data for any purpose other than conducting the study. These restrictions do not apply to information where it is necessary to disclose in confidence to HREC solely for the evaluation of the study.

10. Dissemination

The investigators will implement a dissemination plan that will include:

- key communication strategies for all stakeholders
- an Open Access publication plan
- authorship requirements and publication standards that align with:
  - NHMRC Australian Code for the Responsible Conduct of Research (http://www.nhmrc.gov.au/guidelines-publications/r39)
  - International Committee of Medical Journal Editors Recommendations for the Conduct, Reporting, Editing, and Publication of Scholarly Work in Medical Journals (http://www.icmje.org/recommendations/browse/roles-and-responsibilities/defining-the-role-of-authors-and-contributors.html).
- Any third-party access to the data collected in this study will only be granted if appropriate ethical approvals have been sought. Full de-identified data sets and statistical codes will only be available by contacting a chief investigator and providing the appropriate ethical approvals.

11. Communication of findings to participants

Findings from the research will be communicated to participants, guardians, and facility staff of the Bushland Health Group through newsletters, email bulletins and publicly available reports. Details on how to access the findings of the research will be made available to residents, guardians, and staff of participating facilities.

If there were incidental findings of note that may have implications for the facility involved and may require action (i.e., clinical care implications), they will also be shared. Importantly, there will not be any re-identifiable participant data at such a point in time. As the data collection is used to determine whether a person has an infection or not (and the type of

infection), we do not envisage a major risk of incidental findings.

12. References

Agarwal, N., Meena, C. S., Raj, B. P., Saini, L., Kumar, A., Gopalakrishnan, N., Kumar, A., Balam, N. B., Alam, T., Kapoor, N. R., & Aggarwal, V. (2021). Indoor air quality improvement in COVID-19 pandemic: Review. *Sustain Cities Soc*, *70*, 102942. https://doi.org/10.1016/j.scs.2021.102942

Australian Government Aged Care Quality and Safety Commission (ACQSC). (2022). *Becoming an approved aged care provider*. https://www.agedcarequality.gov.au/providers/becoming-approved-aged-care-provider

Australian Institute of Health and Welfare (AIHW). (2021). *Older Australians*. https://www.aihw.gov.au/reports/older-people/older-australians/contents/demographic-profile

Branche, A. R., & Falsey, A. R. (2015). Respiratory Syncytial Virus Infection in Older Adults: An Under-Recognized Problem. *Drugs & Aging*, *32*(4), 261-269. https://doi.org/10.1007/s40266-015-0258-9

(

)

Brankston, G., Gitterman, L., Hirji, Z., Lemieux, C., & Gardam, M. (2007). Transmission of influenza A in human beings. *The Lancet infectious diseases*, *7*(4), 257-265.

Buising, K. L., Schofield, R., Irving, L., Keywood, M., Stevens, A., Keogh, N., Skidmore, G., Wadlow, I., Kevin, K., & Rismanchi, B. (2022). Use of portable air cleaners to reduce aerosol transmission on a hospital coronavirus disease 2019 (COVID-19) ward. *Infection Control & Hospital Epidemiology*, *43*(8), 987-992.

Burgmann, S., & Janoske, U. (2021). Transmission and reduction of aerosols in classrooms using air purifier systems. *Physics of Fluids*, *33*(3), 033321. https://doi.org/10.1063/5.0044046

Childs, A., Zullo, A. R., Joyce, N. R., McConeghy, K. W., van Aalst, R., Moyo, P., Bosco, E., Mor, V., & Gravenstein, S. (2019). The burden of respiratory infections among older adults in long-term care: a systematic review. *BMC Geriatrics*, *19*(1). https://doi.org/10.1186/s12877-019-1236-6

Commonwealth Department of Health and Aged Care. (2015). *Informed consent*. https://www.australianclinicaltrials.gov.au/how-be-part-clinical-trial/informed-consent

Department of Health Government of Western Australia. (2022). *Communicable Disease Control Directorate Guideline: Use of Air Purifiers*

in WA Healthcare Facilities,. Retrieved from https://ww2.health.wa.gov.au/~/media/Corp/Documents/Health-for/Infectious-disease/HISWA/Air-Purifiers-in-WA-HCFs.pdf

Elliot, A. J., & Fleming, D. M. (2008). Influenza and respiratory syncytial virus in the elderly. *Expert review of vaccines*, *7*(2), 249-258.

European Centre for Disease Prevention and Control (ECDC). (2018). *COMMISSION IMPLEMENTING*

DECISION (EU) 2018/945 of 22 June 2018 on the communicable diseases and related special health issues to be covered by epidemiological surveillance as well as relevant case definitions. Retrieved from https://eur-lex.europa.eu/legal-content/EN/TXT/PDF/?uri=CELEX:32018D0945&from=EN#page=24

Folstein, M. F., Folstein, S. E., & McHugh, P. R. (1975). “Mini-mental state”: a practical method for grading the cognitive state of patients for the clinician. *Journal of psychiatric research*, *12*(3), 189-198.

Folstein, M. F., Robins, L. N., & Helzer, J. E. (1983). The mini-mental state examination. *Archives of general psychiatry*, *40*(7), 812-812.

Greenhalgh, T., Jimenez, J. L., Prather, K. A., Tufekci, Z., Fisman, D., & Schooley, R. (2021). Ten scientific reasons in support of airborne transmission of SARS-CoV-2. *The lancet*, *397*(10285), 1603-1605.

Hoffmann, T., Glasziou, P., Boutron, I., Milne, R., Perera, R., Moher, D., Altman, D., Barbour, V., Macdonald, H., Johnston, M., Lamb, S., Dixon-Woods, M., McCulloch, P., Wyatt, J., Chan, A., & Michie, S. (2014). Better reporting of interventions: template for intervention description and replication (TIDieR) checklist and guide. *Bmj*. https://doi.org/https://doi.org/10.1136/bmj.g1687

Information and Privacy Commission (IPC). (2021). *Guide - Privacy and persons with reduced decision-making capacity* https://www.ipc.nsw.gov.au/guide-privacy-and-persons-reduced-decision-making-capacity#:~:text=An%20%E2%80%9Cauthorised%20representative%E2%80%9D%20acts%20as,enduring%20power%20of%20attorney%2C%20or

Jaul, E., & Barron, J. (2017). Age-related diseases and clinical and public health implications for the 85 years old and over population. *Frontiers in public health*, *5*, 335.

Leung, N. H. L., Chu, D. K. W., Shiu, E. Y. C., Chan, K. H., McDevitt, J. J., Hau, B. J. P., Yen, H. L., Li, Y., Ip, D. K. M., Peiris, J. S. M., Seto, W. H., Leung, G. M., Milton, D. K., & Cowling, B. J. (2020). Respiratory virus shedding in exhaled breath and efficacy of face masks. *Nature Medicine*, *26*(5), 676-680. https://doi.org/10.1038/s41591-020-0843-2

McElhaney, J. E., Zhou, X., Talbot, H. K., Soethout, E., Bleackley, R. C., Granville, D. J., & Pawelec, G. (2012). The unmet need in the elderly: how immunosenescence, CMV infection, co-morbidities and frailty are a challenge for the development of more effective influenza vaccines. *Vaccine*, *30*(12), 2060-2067.

National Health and Medical Research Council (NHMRC), Australian Research Council (ARC), & Universities Australia (UA). (2018a). *Australian Code for the Responsible Conduct of Research*,.

https://www.nhmrc.gov.au/about-us/publications/australian-code-responsible-conduct-research-2018#block-views-block-file-attachments-content-block-1

National Health and Medical Research Council (NHMRC), Australian Research Council (ARC), & Universities Australia (UA). (2018b). *National Statement on Ethical Conduct in Human Research (2007) - Updated 2018*. https://www.nhmrc.gov.au/about-us/publications/national-statement-ethical-conduct-human-research-2007-updated-2018

National Health and Medical Research Council (NHMRC), Australian Research Council (ARC), & Universities Australia (UA). (2019). *Management of Data and Information in Research: A guide supporting the Australian Code for the Responsible Conduct of Research*. Retrieved from https://www.nhmrc.gov.au/about-us/publications/australian-code-responsible-conduct-research-2018

Reddy, M., Heidarinejad, M., Stephens, B., & Rubinstein, I. (2021). Adequate indoor air quality in nursing homes: An unmet medical need. *Science of The Total Environment*, *765*, 144273.

Shi, T., Denouel, A., Tietjen, A. K., Campbell, I., Moran, E., Li, X., Campbell, H., Demont, C., Nyawanda, B. O., Chu, H. Y., Stoszek, S. K., Krishnan, A., Openshaw, P., Falsey, A. R., Nair, H., & Investigators., R. (2020). Global Disease Burden Estimates of Respiratory Syncytial Virus–Associated Acute Respiratory Infection in Older Adults in 2015: A Systematic Review and Meta-Analysis. *The Journal of infectious diseases*, *222*(Supplement_7), S577-S583. https://doi.org/10.1093/infdis/jiz059

Shiu, E. Y., Leung, N. H., & Cowling, B. J. (2019). Controversy around airborne versus droplet transmission of respiratory viruses: implication for infection prevention. *Current opinion in infectious diseases*, *32*(4), 372-379.

Tang, J. W., Marr, L. C., Li, Y., & Dancer, S. J. (2021). Covid-19 has redefined airborne transmission. In (Vol. 373): British Medical Journal Publishing Group.

Tellier, R. (2006). Review of aerosol transmission of influenza A virus. *Emerging infectious diseases*, *12*(11), 1657.

Tellier, R., Li, Y., Cowling, B. J., & Tang, J. W. (2019). Recognition of aerosol transmission of infectious agents: a commentary. *BMC Infectious Diseases*, *19*(1). https://doi.org/10.1186/s12879-019-3707-y

The University of Newcastle (UON). (2017). *Research Data and Primary materials Management Procedure*. https://policies.newcastle.edu.au/document/view-current.php?id=72

Thompson, W. W., Shay, D. K., Weintraub, E., Brammer, L., Cox, N., Anderson, L. J., & Fukuda, K. (2003). Mortality associated with influenza and respiratory syncytial virus in the United States.

Jama, *289*(2), 179-186.

University of Newcastle (UON). (2017). *Research Data and Primary Materials Management Procedure*. https://policies.newcastle.edu.au/document/view-current.php?id=72

van Asten, L., van den Wijngaard, C., van Pelt, W., van de Kassteele, J., Meijer, A., van der Hoek, W., Kretzschmar, M., & Koopmans, M. (2012). Mortality Attributable to 9 Common Infections: Significant Effect of Influenza A, Respiratory Syncytial Virus, Influenza B, Norovirus, and Parainfluenza in Elderly Persons. *The Journal of infectious diseases*, *206*(5), 628-639. https://doi.org/10.1093/infdis/jis415

Wang, G. C., & Casolaro, V. (2014). Immunologic changes in frail older adults.

World Health Organization (WHO) Regional Office for Europe. (2011). WHO Regional Office for Europe guidance for sentinel influenza surveillance in humans.
