# Supplementary material for: Air Purifiers and Acute Respiratory Infections in Residential Aged Care: A Randomized Clinical Trial
Source: JAMA Netw Open. 2024 Nov 11;7(11):e2443769. doi: 10.1001/jamanetworkopen.2024.43769 (PMC11555545; doi:10.1001/jamanetworkopen.2024.43769)
Supplement: Supplement 2. — eTable 1. Fortnightly Routine Data Collection Related to an ARI eAppendix 1. Additional Trial Information eTable 2. Data Related to the Mobility Pattern of Study Participants Over the 2 Phases of the Trial eTable 3. Allocation of Participants by Study Sites eTable 4. Types of Acute Respiratory Tract Infection by Study Period and Intervention Allocation eTable 5. Completion Status by Intervention Allocation eTable 6. Outcomes for Participants Who Dropped Out of the Study eTable 7. Study Location and Outcomes From Participants Who Completed the Study eAppendix 2. Secondary Outcomes eTable 8. Risk of Infections—Cox Proportional Hazards Regressions for Complete and all Available Cases of Infections eTable 9. Mean Days to First Infection eTable 10. Other Outcomes in Participants Who Experienced an ARI eAppendix 3. Sensitivity Analysis eTable 11. Risk of Infections, Phase 1 and 2 Separately eTable 12. Risk of Infections—Cox Proportional Hazard Regressions for Complete and all Available Cases of Infections eTable 13. Risk of Infection, Phase 1—Cox Proportional Hazard Regressions for Complete and All Available Cases of Infections eAppendix 4. Multiple Imputation Model eTable 14. Risk of Acute Respiratory Tract Infection—Logistic Regression Models Without Multivariate Adjustments for the Multiple Imputation Model eTable 15. Risk of Acute Respiratory Tract Infection—Logistic Regression Models eTable 16. Frequency and Percentage of Acute Respiratory Tract Infection by Phase, Intervention, and Site Among Study Completers eFigure 1. Proportion of Infections by Phase, Intervention, and Site for Completers eFigure 2. Survival Curves for Infections, Stratified by Treatment and Phase eAppendix 5. Considerations for Future Studies eReferences [file jamanetwopen-e2443769-s002.pdf]

Supplementary Online Content

Thottiyil Sultanmuhammed Abdul Khadar B, Sim J, McDonald VM, McDonagh J, Clapham M, Mitchell BG. Air purifiers and acute respiratory infections in residential aged care: a crossover randomized clinical trial. *JAMA Netw Open*. 2024;7(11):e2443769. doi:10.1001/jamanetworkopen.2024.43769

eTable 1. Fortnightly Routine Data Collection Related to an ARI
eAppendix 1. Additional Trial Information
eTable 2. Data Related to the Mobility Pattern of Study Participants Over the 2 Phases of the Trial
eTable 3. Allocation of Participants by Study Sites
eTable 4. Types of Acute Respiratory Tract Infection by Study Period and Intervention Allocation
eTable 5. Completion Status by Intervention Allocation
eTable 6. Outcomes for Participants Who Dropped Out of the Study
eTable 7. Study Location and Outcomes From Participants Who Completed the Study
eAppendix 2. Secondary Outcomes
eTable 8. Risk of Infections—Cox Proportional Hazards Regressions for Complete and all Available Cases of Infections
eTable 9. Mean Days to First Infection
eTable 10. Other Outcomes in Participants Who Experienced an ARI
eAppendix 3. Sensitivity Analysis
eTable 11. Risk of Infections, Phase 1 and 2 Separately
eTable 12. Risk of Infections—Cox Proportional Hazard Regressions for Complete and all Available Cases of Infections
eTable 13. Risk of Infection, Phase 1—Cox Proportional Hazard Regressions for Complete and All Available Cases of Infections
eAppendix 4. Multiple Imputation Model
eTable 14. Risk of Acute Respiratory Tract Infection—Logistic Regression Models Without Multivariate Adjustments for the Multiple Imputation Model
eTable 15. Risk of Acute Respiratory Tract Infection—Logistic Regression Models
eTable 16. Frequency and Percentage of Acute Respiratory Tract Infection by Phase, Intervention, and Site Among Study Completers
eFigure 1. Proportion of Infections by Phase, Intervention, and Site for Completers
eFigure 2. Survival Curves for Infections, Stratified by Treatment and Phase
eAppendix 5. Considerations for Future Studies
eReferences

This supplementary material has been provided by the authors to give readers additional information about their work.

© 2024 Thottiyil Sultanmuhammed Abdul Khadar B et al. *JAMA Network Open*.

Additional trial information

Overview of data collected

eTable 1: Fortnightly routine data collection related to an ARI

Data Item	Source of data	Data Extraction
Signs and symptoms of a [1]ARI with the date of onset of the first symptom.<br>cough<br>sore throat<br>shortness of breath<br>coryza<br>AND<br>clinician's judgment that the illness is due to an infection	Data collated by reviewing:<br>Participants' medical record<br>RACF clinical record.<br>Patient health summary from [5]GP<br>[13]RACF nursing records<br>Verbal reports from:<br>[14]RNs<br>Participants/legal guardian	[15]Manual data entry
[2]RAT performed (Y/N)	Data collated by reviewing:<br>Participants' medical record<br>[13]RACF clinical record<br>Patient health summary from [5]GP<br>[13]RACF nursing records	[15]Manual data entry
[2]RAT results (if any).	Data collated by reviewing:<br>Participants' medical record<br>Patient health summary from [5]GP<br>Hospital discharge summary (if any)<br>[13]RACF nursing records<br>Laboratory records	[15]Manual data entry
Swab sent, ([3]RT-PCR)/culture/[4]SE), (Y/N)	Data collated by reviewing:<br>Participants' medical record<br>[13]RACF clinical record<br>Patient health summary from [5]GP<br>[13]RACF nursing records	[15]Manual data entry
Lab Results, ([3]RT-PCR)/culture/[4]SE), (if any).	Data collated by reviewing:<br>Participants' medical record<br>Patient health summary from GP<br>Hospital discharge summary (if any)<br>[13]RACF nursing records<br>Laboratory records	[15]Manual data entry

© 2024 Thottiyil Sultanmuhammed Abdul Khadar B et al. *JAMA Network Open*.

Data Item (Contd.)	Source of data (Contd.)	Data Extraction (Contd.)
Medical consultations[7] ([5]GP and/or [6]NP) due to a [1]ARI	Data collated by reviewing:<br>Participants' medical record<br>Patient health summary from [5]GP<br>[3]RACF nursing records	[15]Manual data entry
The number of [9]visits to an [8]ED due to a [1]ARI	Data collated by reviewing:<br>Participants' medical record<br>Patient health summary from [5]GP<br>Hospital discharge summary (if any)<br>[3]RACF nursing records	[15]Manual data entry
The number of [10] hospital admissions due to a [1]ARI	Data collated by reviewing:<br>Participants' medical record<br>Patient health summary from [5]GP<br>Hospital discharge summary (if any)<br>[3]RACF nursing records	[15]Manual data entry
The participants' documented mobility status,	Data collated from:<br>Review of [3]RACF electronic clinical nursing records<br>The participants' current mobility assessments and care plans.<br>Verbal report from [14]RNs<br>Review of physiotherapist's assessments<br>Verbal reports from physiotherapists	[15]Manual data entry
[11] Changes to specific medications	Data collated by reviewing current:<br>Patient health summary from [5]GP<br>Medication chart<br>Hospital discharge summary (if any)	[15]Manual data entry
[12]Update of vaccinations	Data collated by reviewing:<br>[3]RACF records<br>Participants' medical record<br>Contacting [5]GP for updates (if required)	[15]Manual data entry

[1]Acute Respiratory Infection, [2]Rapid Antigen Test, [3]Real Time-Polymerase Chain Reaction, [4]Serum Examination, [5]General Practitioner, [6]Nurse Practitioner, [7] Including the date of each medical consultation, [8]Emergency Department, [9]Including the date of each ED visit, [10]Including the date of each hospital admission, [11]cytotoxic, antibiotics, and antivirals, [12] any additional vaccinations received, [13] Residential Aged Care Facility, [14] Registered Nurses, [15]on to the online form using an iPad by visiting RACF

© 2024 Thottiyil Sultanmuhammed Abdul Khadar B et al. *JAMA Network Open*.

eAppendix 1. Additional Trial Information

Additional information regarding the intervention

There are different types of HEPA filters, a HEPA-14 filter is considered to have an efficiency of trapping ≥ 99.995% of particles to 0.3µm in size.[1] The air purifiers used in the trial did not use ionization as a form of air purification. To maintain the integrity of the air purifier, 'do not open' stickers were affixed to the door seal on either side of the air purifier to detect any tampering with the device. The procedure of installing the air purifiers in participants' rooms was mutually agreed upon between the research team and the participant or their next of kin. The pre-filters of the air purifiers were regularly cleaned during the trial as per the manufacturer's instructions by a person not involved in the data collection process.

We followed the manufacturer's instructions for cleaning pre-filters and changing HEPA filters to enhance and maintain the filtering capacity of air purifiers. We also used personal protective equipment while cleaning and changing pre-filters and HEPA filters.

Additional information on data collection procedures

During routine data collection, the researcher visited the participants and screened the medical, nursing, and laboratory records of all participants for any evidence of an ARI. Where an ARI was determined, the data were collected based on the details related to the ARI. The processes for collecting these fortnightly routine data and the details of the data collected are presented in eTable 1.

The pattern of mobility data included time spent in the dining room, activities room, or in other social activities and aimed to capture a baseline of time spent out of their room as part of regular or routine activities. This was then quantified into an estimate of hours spent outside the room on a 'typical' day.

Additional results

Baseline and other demographic data

eTable 2. Data related to the mobility pattern of study participants over the two phases of the trial

Variables	Completers (n/%) n=104	Non-completers (n/%) n=31	All participants (n/%) n=135
Mobility Data: Time spent inside the room (in hours)			
**Phase 1**			
8-12	5(4.8)	1(3.2)	6(4.4)
12-18	25(24.0)	11(35.5)	36(26.7)
18-21	33(31.7)	12(38.7)	45(33.3)
21-24	31(29.8)	6(19.4)	37(27.4)
24	10(9.6)	24(77.4)	34(25.2)
**Phase 2**			
8-12	5(4.8)	1(3.2)	6(4.4)
12-18	26(25.0)	4(12.9)	30(22.2)
18-21	30(28.9)	3(9.7)	33(24.4)
21-24	31(29.8)	0(0.0)	31(23.0)
24	12(11.5)	1(3.2)	13(9.6)
NA	NA	22(71.0)	22(16.3)

Note. n= number of participants

© 2024 Thottiyil Sultanmuhammed Abdul Khadar B et al. *JAMA Network Open*.

Allocation of participants by study sites

eTable 3. Allocation of participants by study sites

Characteristic	Control-Intervention n/N (%) (N=65)	Intervention-Control n/N (%) (N=70)	Overall n/N (%) (N=135)
**Study sites**			
A	26/65 (40)	27/70 (39)	53/135 (39)
B	10/65 (15)	15/70 (21)	25/135 (19)
C	29/65 (45)	28/70 (40)	57/135 (42)

Note: N=Total number of participants, n= number of participants in the specific group

© 2024 Thottiyil Sultanmuhammed Abdul Khadar B et al. *JAMA Network Open*.

Primary outcome

eTable 4. Types of acute respiratory tract infection by study period and intervention allocation (n=73)

Study period	Control (n)	Intervention (n)	All (n)
**Phase 1**			
SARS-CoV-2	11	7	18
Influenza (A or B)	0	0	0
Respiratory syncytial virus	10	4	14
Rhinovirus	0	0	0
Unknown	8	10	18
ARI – no pathology	5	5	10
**Phase 2**			
SARS-CoV-2	1	0	1
Influenza (A or B)	0	0	0
Respiratory syncytial virus	1	1	2
Rhinovirus	0	1	1
Unknown	11	8	19
ARI – no pathology	12	8	20

Note: n=number of participants

© 2024 Thottiyil Sultanmuhammed Abdul Khadar B et al. *JAMA Network Open*.

Completion status

among participants receiving the intervention vs the control

eTable 5. Completion status by intervention allocation

Study completion status	Control-Intervention n/N (%) (N=65)	Intervention-Control n/N (%) (N=70)	Overall n/N (%) (N=135)
Completed	50/65 (77)	54/70 (77)	104/135 (77)
Not completed	15/65 (23)	16/70 (23)	31/135 (23)

Note: N=Total number of participants, n= number of participants in the specific group

© 2024 Thottiyil Sultanmuhammed Abdul Khadar B et al. *JAMA Network Open*.

Outcomes for participants who dropped out

eTable 6. Outcomes for participants who dropped out of the study

	With ARI (n)		No ARI (n)		
	**Control-Intervention**	**Intervention-Control**	**Control-Intervention**	**Intervention-Control**	**Overall (n)**
**Phase 1**	2	6	8	6	22
**Phase 2**	0	1	4	4	9
**Overall**	2	7	12	10	31

Note: n= number of participants

© 2024 Thottiyil Sultanmuhammed Abdul Khadar B et al. *JAMA Network Open*.

eTable 7. Study location and outcomes from participants who completed the study.

Characteristic	Control-Intervention n/N (%)	Intervention-Control n/N (%)	Overall n/N (%)
**ARIs**	33/100 (33)	29/108 (27)	62/208 (30)
**Sites**			
**A**	20/50 (40)	21/54 (39)	41/104 (39)
**B**	7/50 (14)	12/54 (22)	19/104 (18)
**C**	23/50 (46)	21/54 (39)	44/104 (42)
**Completed study**	50/50 (100)	54/54 (100)	104/104 (100)

Note: ARIs=Acute Respiratory Infections; N=Total number of participants, n= number of participants in the specific group

© 2024 Thottiyil Sultanmuhammed Abdul Khadar B et al. *JAMA Network Open*.

eAppendix 2

Secondary outcomes

Time to first infection
Mean days to the first infection have been illustrated (eTable 9). A key assumption in Cox proportional hazard modelling is that the ratio of the hazard of two individuals is constant over time. The low ARI rates make formal tests of these assumptions difficult, there were 4 ARIs in phase 2 after 50 days. The eTable 13The represents the Cox proportional hazard model for phase 1 only. The results were similar to the full model (HR 0.67, (95%CI 0.42, 1.07, p=0.090) (eTable 12) and for phase 1 (HR 0.61, (95%CI 0.35 1.08, p=0.089) eTable 13. The hazard ratio from the full model can be interpreted as the average HR over the trial period (eTable 12 and eTable 13). eTable 16 represents the proportion of infections by phase, intervention, and site for completers, and a visual cue of the site driving these results has been illustrated in eFigure1. Site B had more infections in the intervention than control.

Secondary analysis – additional information

Time to first infection was assessed with Cox proportional hazard model with mixed effects (Frailty model). Fixed effects included intervention, with design-specific variables for each phase and facility. The random effect in the model was the unique participant identification number to account for within-person correlation. A Cox proportional hazard model for phase 1 only was assessed for reference. Hazard ratios with 95% confidence intervals and p-values were calculated. This analysis was conducted for all available cases and a sub-group analysis of participants who completed the study. For the completer's sub-group, participants with no infection were censored at the endpoint of each phase. For all available case groups, participants with no infection were censored at the endpoint of each phase or their dropout time point, and participants who dropped out with an infection were included as an event with time to their infection. Kaplan-Meier survival plots stratified by intervention and stratified by phase and intervention were calculated. The restricted mean time to infections with standard errors was calculated.

© 2024 Thottiyil Sultanmuhammed Abdul Khadar B et al. *JAMA Network Open*.

eTable 8. Risk of infections, Cox proportional hazard regressions for complete and all available cases for infections

Characteristic	Completed Study (N=104)			All available (N=135)		
	HR	95% CI	p-value	HR	95% CI	p-value
**Intervention**	0.62	0.37 to 1.02	0.061	0.67	0.42 to 1.07	0.090
**Phase**						
1	—	—		—	—	
2	0.55	0.32 to 0.92	0.024	0.53	0.32 to 0.87	0.013
**Sites**						
A	—	—		—	—	
B	1.26	0.66 to 2.42	0.484	1.16	0.63 to 2.15	0.638
C	0.81	0.46 to 1.43	0.465	0.93	0.55 to 1.57	0.786

Note: HR = Hazard Ratio; CI = Confidence Interval; N = number of participants

© 2024 Thottiyil Sultanmuhammed Abdul Khadar B et al. *JAMA Network Open*.

eTable 9. Mean days to first infection (all participants, n=135)

Characteristic	Restricted mean (days)	
	Mean (90days)	SE
**Overall**		
Control	74.20	2.27
Intervention	78.14	2.13
**Phase 1**		
Control	72.87	2.63
Intervention	78.78	2.37
**Phase 2**		
Control	75.85	3.75
Intervention	77.27	3.77

Note: SE= Standard Error

© 2024 Thottiyil Sultanmuhammed Abdul Khadar B et al. *JAMA Network Open*.

Other outcomes

eTable 10. Other outcomes in participants who experienced an ARI (n=73)

Outcomes	Phase 1 Intervention n (%)	Phase 1 Control n (%)	Phase 2 Intervention n (%)	Phase 2 Control n (%)	Overall n (%)
Number of medical consultations	12(16.44)	27(36.99)	9(12.33)	7(9.59)	55(75.35)
Number of ED presentations	1(1.37)	5(6.85)	4(5.48)	0	10(13.70)
Number of hospital admissions	1(1.37)	5(6.85)	4(5.48)	0	10(13.70)

Note: n = number of participants

© 2024 Thottiyil Sultanmuhammed Abdul Khadar B et al. *JAMA Network Open*.

eAppendix 3

Sensitivity analysis

The effect of the intervention was less in this sensitivity analysis, (OR 0.65, 95%CI 0.37 to 1.16, p=0.145). Additional sensitivity analyses are presented (eTable 12 and eTable 13). eTable 12 presents the risk of acute respiratory infections for phases 1 and 2 separately, and eTable 13 for Phase 1 only.

© 2024 Thottiyil Sultanmuhammed Abdul Khadar B et al. *JAMA Network Open*.

eTable 11. Risk of infections Phase 1 and 2 separately complete case

Characteristic	Phase 1			Phase 2		
	OR	95% CI	p-value	OR	95% CI	p-value
**Treatment**						
Control	—	—		—	—	
Intervention	0.46	0.20 to 1.03	0.060	0.78	0.28 to 2.09	0.622
**Sites**						
A	—	—		—	—	
B	0.64	0.19 to 2.01	0.450	2.92	0.88 to 9.94	0.080
C	0.70	0.29 to 1.70	0.435	0.66	0.20 to 2.08	0.476
Number of observations	104			104		

Note: OR = Odds Ratio; CI = Confidence Interval

© 2024 Thottiyil Sultanmuhammed Abdul Khadar B et al. *JAMA Network Open*.

eTable 12. Risk of infections, Cox proportional hazard regressions for complete and all available cases for infections

Characteristic	Completed Study (N=104)			All available (N=135)		
	**HR**	**95% CI**	**p-value**	**HR**	**95% CI**	**p-value**
**Intervention**	0.62	0.37 to 1.02	0.061	0.67	0.42 to 1.07	0.090
**Phase**						
1	—	—		—	—	
2	0.55	0.32 to 0.92	0.024	0.53	0.32 to 0.87	0.013
**Sites**						
A	—	—		—	—	
B	1.26	0.66 to 2.42	0.484	1.16	0.63 to 2.15	0.638
C	0.81	0.46 to 1.43	0.465	0.93	0.55 to 1.57	0.786

Note: HR = Hazard Ratio; CI = Confidence Interval; N = number of participants

© 2024 Thottiyil Sultanmuhammed Abdul Khadar B et al. *JAMA Network Open*.

eTable 13. Risk of infection phase 1, Cox proportional hazard regressions for complete and all available cases for infections

Characteristic	Completed Study (N=104)			All available (N=135)		
	HR	95% CI	p-value	HR	95% CI	p-value
**Intervention**	0.55	0.29 to 1.03	0.062	0.61	0.35 to 1.08	0.089
**Sites**						
A	—	—		—	—	
B	0.76	0.30 to1.93	0.566	0.68	0.29 to 1.59	0.371
C	0.93	0.47 to 1.82	0.828	1.10	0.60 to 2.00	0.753

Note: HR = Hazard Ratio; CI = Confidence Interval

© 2024 Thottiyil Sultanmuhammed Abdul Khadar B et al. *JAMA Network Open*.

eAppendix 4

Additional information: Multiple Imputation model

Since there was a need to account for missing data, primarily because of participants dying during the study. Previous studies highlighted the requirement of managing the missing data in research involving people in palliative care settings.[2] Although our study involved residents of RACFs, the risk of mortality in this cohort is similar. We attempted to account for missing data using multiple imputations, which indicated non-statistically significant results (OR 0.56, 95%CI 0.30 to 1.03, p=0.062) (Table 3). However, it could be argued this is a clinically important reduction.[3,4]. The analysis using the multiple imputation model has been shown (eTable 14 and eTable 15).

© 2024 Thottiyil Sultanmuhammed Abdul Khadar B et al. *JAMA Network Open*.

eTable 14: Risk of acute respiratory tract infection: logistic regression models without multivariate adjustments for the multiple imputation model

Characteristic	All participants			Multiple Imputation			Completers		
	OR	95% CI	p-value	OR	95% CI	p-value	OR	95% CI	p-value
**Treatment**									
Control	—	—		—	—		—	—	
Intervention	0.57	0.32 to 1.04	0.069	0.65	0.37 to 1.16	0.145	0.53	0.28 to 1.00	0.048
**Phase**									
1	—	—		—	—		—	—	
2	0.37	0.20 to 0.69	0.002	0.60	0.30 to 1.19	0.144	0.40	0.21 to 0.76	0.006
**Sites**									
A	—	—		—	—		—	—	
B	1.24	0.54 to 2.82	0.611	1.02	0.45 to 2.29	0.962	1.29	0.55 to 3.05	0.556
C	0.84	0.43 to 1.65	0.607	0.81	0.42 to 1.56	0.528	0.70	0.34 to 1.42	0.319
**Number of observations**	226						208		

Note: MI = Multiple imputation (N=100 imputations, N=135 participants per phase); OR = Odds Ratio; CI = Confidence Interval; Phase 1 & 2 (N=104 participants); Phase 1 (N=121 participants); Phase 2 (N=105 participants)

© 2024 Thottiyil Sultanmuhammed Abdul Khadar B et al. *JAMA Network Open*.

eTable 15. Risk of acute respiratory tract infection: logistic regression models

Characteristic	All participants			Multiple Imputation			Completers		
	OR	95% CI	p-value	OR	95% CI	p-value	OR	95% CI	p-value
**Treatment**									
Control	—	—		—	—		—	—	
Intervention	0.57	0.32 to 1.04	0.069	0.56	0.30 to 1.03	0.062	0.53	0.28 to 1.00	0.048
**Phases**									
1	—	—		—	—		—	—	
2	0.37	0.20 to 0.69	0.002	0.42	0.21 to 0.86	0.018	0.40	0.21 to 0.76	0.006
**Sites**									
A	—	—		—	—		—	—	
B	1.24	0.54 to 2.82	0.611	1.19	0.51 to 2.79	0.688	1.29	0.55 to 3.05	0.556
C	0.84	0.43 to 1.65	0.607	0.85	0.42 to 1.72	0.655	0.70	0.34 to 1.42	0.319
**Number of observations**	226						208		

Note: MI = Multiple Imputation (N=100 imputations; N=135 participants per phase); OR = Odds Ratio; CI = Confidence Interval; Phase 1 & 2 (N=104 participants); Phase 1 (N=121 participants); Phase 2 (N=105 participants).

© 2024 Thottiyil Sultanmuhammed Abdul Khadar B et al. *JAMA Network Open*.

eTable 16. Frequency and percentage of acute respiratory tract infection by phase, intervention, and site among study completers

Site	Control Phase 1	Intervention Phase 1	Control Phase 2	Intervention Phase 2
A, no. (%), (n=41)	11/20(55.0)	7/21(33.3)	6/21(28.6)	2/20(10.0)
B, no. (%), (n=19)	2/7(28.6)	4/12(33.3)	4/12(33.3)	4/7(57.1)
C, no. (%), (n=44)	11/23(47.8)	5/21(23.8)	3/21(14.3)	3/23(13.0)

Note: n = number of participants

© 2024 Thottiyil Sultanmuhammed Abdul Khadar B et al. *JAMA Network Open*.

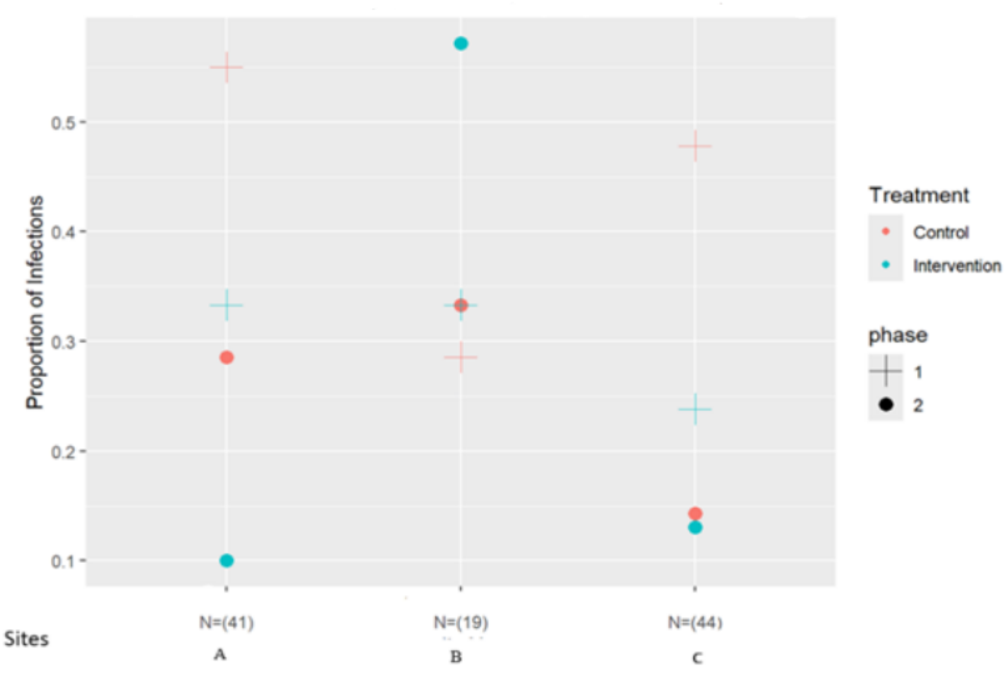

eFigure1. Proportion of infections by phase, intervention, and site for completers

Legend: A visual cue that illustrates the proportion of infections by phase, interventions, and site for completers. Phase 2 was driven by site B and had more infections than control.

© 2024 Thottiyil Sultanmuhammed Abdul Khadar B et al. *JAMA Network Open*.

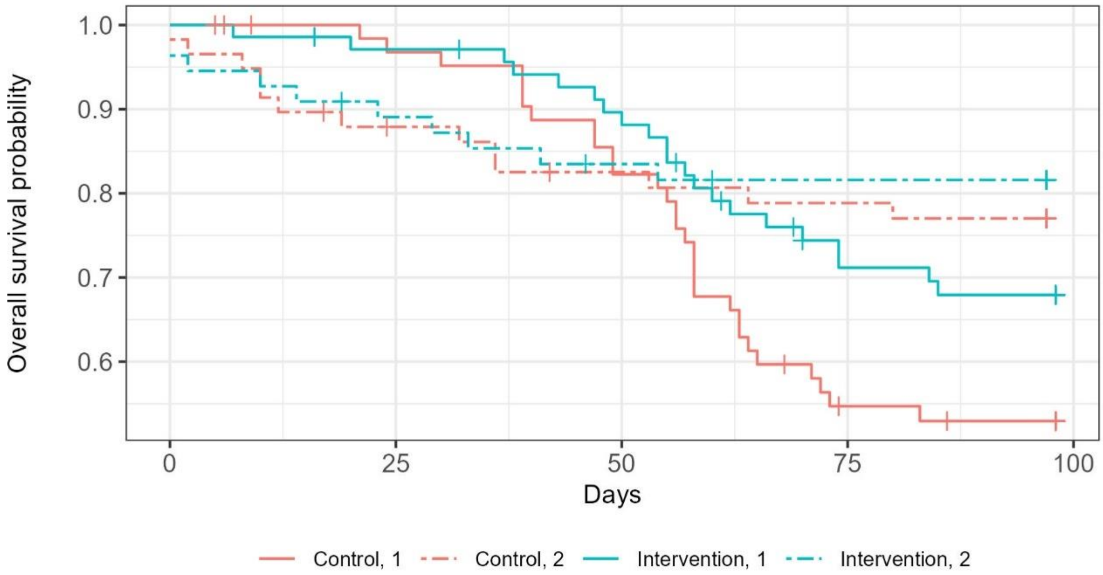

At Risk

	0	25	50	75	100
Control, 1	65	60	51	31	0
Control, 2	58	49	45	43	0
Intervention, 1	70	66	60	44	0
Intervention, 2	55	48	44	42	0

Events

	0	25	50	75	100
Control, 1	0	2	11	28	29
Control, 2	1	7	10	12	13
Intervention, 1	0	2	8	19	21
Intervention, 2	2	6	9	10	10

Note: Control 1=control group Phase 1; Control 2=control group Phase 2
Intervention 1= Intervention group Phase 1; intervention 2= Intervention group Phase 2

eFigure 2. Survival curves for infections stratified by treatment and phase
Legend: eFigure 2 presents the time to infection survival curve stratified by treatment and phase. The probability of an infection (survival probability) of the control group dropped sooner than the intervention group indicating that the time to infection was shorter for the control group than the intervention. The difference between the survival curves was larger in Phase 1 than in Phase 2.

© 2024 Thottiyil Sultanmuhammed Abdul Khadar B et al. *JAMA Network Open*.

eAppendix 5

Considerations for future studies

To enhance the treatment outcomes and gain a deeper understanding of the relationship between illness and its effects, a recent study on COVID-19[5] suggests evaluating frailty in older adults. Future studies could collect data on frailty, to improve the understanding of its relationship with respect to ARI and interventions such as air purification.

© 2024 Thottiyil Sultanmuhammed Abdul Khadar B et al. *JAMA Network Open*.

eReferences

1. European Standards. EN 1822-1High efficiency air filters (EPA, HEPA and ULPA) - Part 1: Classification, performance testing, marking. 2019.
2. Hussain JA, White IR, Johnson MJ, et al. Development of guidelines to reduce, handle and report missing data in palliative care trials: a multi-stakeholder modified nominal group technique. *Palliative medicine* 2022; **36**(1): 59-70.
3. Ranganathan P, Pramesh C, Buyse M. Common pitfalls in statistical analysis: Clinical versus statistical significance. Perspect Clin Res 2015; 6 (3): 169-70.
4. McShane BB, Gal D, Gelman A, Robert C, Tackett JL. Abandon statistical significance. *The American Statistician* 2019; **73**(sup1): 235-45.
5. Hussien H, Nastasa A, Apetrii M, Nistor I, Petrovic M, Covic A. Different aspects of frailty and COVID-19: points to consider in the current pandemic and future ones. *BMC geriatrics* 2021; **21**: 1-11.

© 2024 Thottiyil Sultanmuhammed Abdul Khadar B et al. *JAMA Network Open*.
