# Supplementary material for: Air Purifiers and Acute Respiratory Infections in Residential Aged Care: A Randomized Clinical Trial
Source: JAMA Netw Open. 2024 Nov 11;7(11):e2443769. doi: 10.1001/jamanetworkopen.2024.43769 (PMC11555545; doi:10.1001/jamanetworkopen.2024.43769)
Supplement: Supplement 3. — Data Sharing Statement [file jamanetwopen-e2443769-s003.pdf]

Data Sharing Statement

Thottiyil Sultanmuhammed Abdul Khadar. Air Purifiers and Acute Respiratory Infections in Residential Aged Care. *JAMA Netw Open*. Published November 11, 2024. doi:10.1001/jamanetworkopen.2024.43769

Data
Additional Information: Australian New Zealand Clinical Trial Registry (https://www.anzctr.org.au); Trial Id: ACTRN12623000347662.
Data available: Yes
Data types: Deidentified participant data
How to access data: Bismi.ThottiyilSultanmuhammedAbdul@uon.edu.au
When available: beginning date: 01-01-2025

Supporting Documents
Document types: None

Additional Information
Who can access the data: researchers whose proposed use of the data has been approved
Types of analyses: for a specified purpose.
Mechanisms of data availability: After ethics approval has been granted for the provision of data
Any additional restrictions: Relevant ethics approvals for sharing any data must be sought and approved prior to sharing any data
